# Constructing mesoscale functionomics by neural dynamics subspace clustering

**DOI:** 10.1093/nsr/nwag420

**Published:** 2026-07-11

**Authors:** Yeyi Cai, Xinhong Xu, Guihua Xiao, Huaming Ling, Zuoqiang Shi, Minghuan Wang, Mingrui Wang, Fenghua Chen, Jiamin Wu, Qionghai Dai

**Affiliations:** Department of Automation, Tsinghua University, Beijing 100084, China; Institute for Brain and Cognitive Sciences, Tsinghua University, Beijing 100084, China; Department of Automation, Tsinghua University, Beijing 100084, China; Institute for Brain and Cognitive Sciences, Tsinghua University, Beijing 100084, China; Department of Automation, Tsinghua University, Beijing 100084, China; Institute for Brain and Cognitive Sciences, Tsinghua University, Beijing 100084, China; Beijing National Research Center for Information Science and Technology, Tsinghua University, Beijing 100084, China; Department of Mathematical Science, Tsinghua University, Beijing 100084, China; Department of Mathematical Science, Tsinghua University, Beijing 100084, China; Department of Neurology, Tongji Hospital, Tongji Medical College, Huazhong University of Science and Technology, Wuhan 430074, China; Tsinghua Shenzhen International Graduate School, Tsinghua University, Shenzhen 518071, China; Institute for Brain and Cognitive Sciences, Tsinghua University, Beijing 100084, China; Department of Automation, Tsinghua University, Beijing 100084, China; Institute for Brain and Cognitive Sciences, Tsinghua University, Beijing 100084, China; IDG/McGovern Institute for Brain Research, Tsinghua University, Beijing 100084, China; Department of Automation, Tsinghua University, Beijing 100084, China; Institute for Brain and Cognitive Sciences, Tsinghua University, Beijing 100084, China; IDG/McGovern Institute for Brain Research, Tsinghua University, Beijing 100084, China

**Keywords:** functionomics, subspace clustering, cortical dynamics

## Abstract

Building a fine-grained functional atlas of the brain is crucial for deciphering bio-intelligence. However, the intricate and non-linear interactions at single-neuron level lead to asynchronous and heterogeneous firing patterns among closely interacting neural populations, thereby challenging correlated-firing-based clustering methods. Here, we present Functional subspace clustering based on sparse Representation of Intrinsic Dynamics (FRID), an unsupervised approach for identifying neurons with shared microcircuit connectivity and information encoding properties (referred to as ‘Functionomics’) from mesoscale neural recordings. FRID significantly outperforms correlated-firing-based methods in both simulated complex networks and empirical calcium recordings. The accuracy and utility of FRID are validated across sensory integration and decision-making tasks to produce functionomic clusters with improved resolution and coding specificity than those defined by anatomical functional areas. As representative findings, we demonstrate single-cell level functional remapping during recovery from ischemic stroke and the learning-induced reorganization of functional modularization. Together with large-scale neural recordings, FRID could serve as a general data-driven paradigm to accelerate the understanding of mesoscale brain functions.

## INTRODUCTION

**Figure 1.**
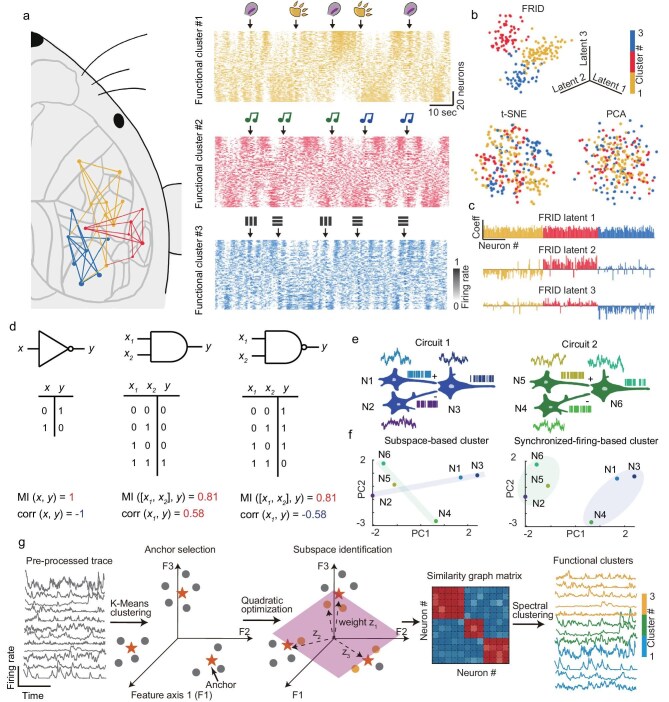
Diagram of unsupervised functional mapping by subspace clustering. (a) Left, illustration of functional architecture revealing diverse modularized neuronal circuits with distinct functions and complex functional connections at single-cell resolution. Right, an example of neural dynamics of different functional clusters encoding different sensory and behavioral variables. Discerption of these clusters helps resolve the functional structure of the brain. (b) Latent space visualization of neurons with FRID, t-SNE and PCA of an example simulated dynamic system. FRID latent axis separates functional modules better than conventional dimension-reduction approaches. (c) The coefficients of neurons in the three FRID latent axis. Each latent axis intuitively represents a mode of affinity with other neurons in the network. (d) Top, gate circuits in electrical circuits as an analogy to neural circuits. Left, NOT gate. Middle, AND gate. Right, exclusive OR gate. Bottom, values of MI and Pearson correlation coefficient of different gate circuits. (e) Two example local neuronal circuits. Each circuit contains three neurons, two of which deliver input to the third neuron. One input neuron has excitatory synapse and the other one inhibitory. (f) The PCs of neural dynamics of the neural circuits shown in panel (e). Subspace-based clustering assumes neurons of the same functional module land on the same subspace (a line in two-dimensional (2D) space), and correlated-firing-based clustering methods assume they land in a local area. In the simulated circuit, subspace-based method achieved a more accurate clustering result. (g) Pipeline of our unsupervised clustering method, named FRID. The K-means clustering method is first applied to extract anchors from the pre-processed traces. After that, the sparse affinity matrix from anchors to all the neural traces is obtained by leveraging constrained quadratic programming with the Frobenius norm penalty. Then the similarity graph matrix of the sparse affinity is calculated. The final clustering result is obtained after applying spectral clustering on the similarity graph matrix.

Bio-intelligence emerges from the collaborative activities of intricately wired neurons [1]. To understand the complex neural network, a natural starting point is the disentanglement of its basic functional modules [2,3]. Functional classification serves as the foundation for elucidating the cross-scale hierarchical organization and information flow in the brain [4]. Meanwhile, neural classification ensures that functional and network analysis can be conducted systematically and reproducibly across different subjects and behavioral protocols. In the past decades, the development of high-throughput neural recording techniques [5,6] has offered a data-driven opportunity to construct this functional map from recorded neural activities. With the help of fluorescent indicators of neural activities [7] and mesoscale single-neuron resolution microscopy [8–10], up to tens of thousands of neurons can now be recorded simultaneously for hours [11,12]. However, how to link these neural activities to their functional counterparts [13] and determine the role that different neurons play in the neural orchestra remains a challenge (Fig. 1a). Manual inspection of single-neuron tuning properties to search for task-related neurons is both laborious and impractical in large-scale neural recording [14]. Therefore, a data-driven approach to reveal functional modules is urgently needed in this unprecedented large-scale recording era.

While neural type classification has long been investigated in terms of genomics, transcriptomics [4,15], anatomy and connectome [16–18], these approaches remain coarse-grained or static. True function of the brain can only be understood *in vivo*. However, in mesoscale neural recording, neurons exhibit highly complex and unpredictable firing patterns, even triggered by the same external stimulus [19]. Additionally, spontaneous activities drive cortex-wide activities that intermingle with functionally related signals [20,21]. Moreover, single-neuron tuning curves fail to encode high-dimensional stimuli, and population activities can only be meaningfully decoded when considered as an entirety [22]. Therefore, neurons considered as isolated individuals cannot be reliably assigned to reasonable functional clusters in large-scale recordings. Some pioneering efforts have been made in categorizing neurons from the perspective of functional specialization. For instance, neurons can be clustered based on similar tuning properties in a supervised manner [23–27]. In addition, non-negative matrix factorization (NMF) has been applied for unsupervised clustering [28]. Moreover, studies combining *in vivo* imaging and transcriptomics have investigated the gene-function correspondence, including state modulation [29,30] and certain task-related features [31,32]. However, these approaches are limited to small neural populations within specific functional brain areas and are insufficient for large-scale data-driven discoveries. Some other proposed clustering methods are designed for spiking data [33–35]. Due to the probability modeling and high temporal resolution demands, these methods are not well suited for large-scale calcium imaging analysis. Essentially, most current clustering methods assume correlated firings within functional clusters, thereby underestimating the heterogeneity and complexity of neuron circuits. Therefore, efficient analysis considering the complex internal dynamics of neural circuits remains an unsolved problem, especially for large-scale single-trial studies.

Here, we present Functional subspace clustering based on sparse Representation of Intrinsic Dynamics (FRID), which performs anchor-guided large-scale subspace clustering on mesoscale neural recording data to identify diverse neuronal circuits with distinct information encoding properties at single-cell resolution (referred to as ‘Functionomics’) without any behavioral supervision. We first validated FRID on a simulated modularized recurrent dynamical network. With known ground truth (GT), FRID successfully recovered the functional modules with ∼98% accuracy from temporal traces when the inter-module connectivity is sparse, significantly outperforming both linear and non-linear correlated-firing-based methods (which performed near random). Then, we applied FRID to empirical layer-2/3 cortex-wide three-dimensional (3D) neural activities of tens of thousands of neurons in mice recorded by the real-time, ultra-large-scale, high-resolution 3D mesoscope (RUSH3D) [11,12] with calcium indicators. We adopted both passive exposure and active engagement tasks, and found that functionomic clusters exhibited anatomically coherent organization, regardless of the specific behavior protocol we chose. In addition, compared with anatomic atlas, FRID identified more task-informative populations than those defined by coarse-grained functional brain areas. We further employed a multi-sensory exposure experiment to examine the sensory information carried by functional clusters. A multi-session FRID extension was designed to disentangle the consistent sensory information conveyed by functional clusters despite the dynamic reorganization of functional architecture. FRID was also applied in a study of ischemic stroke, revealing the remapping of cellular network functions during recovery. Finally, we applied FRID to a cross-day recording of Go/No-Go protocol. We observed the modulation of functional organization by long-term learning and short-term state shift. Altogether, we believe FRID serves as a generalizable framework for functional classification of neurons and construction of dynamically organized cellular functional map, thereby accelerating data-driven discoveries in the field of large-scale neural recording.

## RESULTS

### Principle of FRID

The difficulty underlying cellular-level encoding functional study is that neurons within a single functional cluster could be highly heterogeneous, forming a high-dimensional neural manifold [36], while neurons from different functional clusters often exhibit global co-fluctuation driven by ongoing body movements [20,21]. Hebb’s law [37] says ‘Fire together, wire together’, suggesting the connections between neuron pairs may be strengthened via correlated neural activities. However, the inverse of this rule does not necessarily hold (Fig. 1a). Neurons wire together do not always fire together (Fig. 1b). A simple analogy could be made by examining the input and the output of the logical gate (Fig. 1d). The Pearson correlation between the input and output signals could be either very low (NOT gate), slightly positive (AND gate) or slightly negative (exclusive OR gate). The linear correlation fails to capture the non-linear relationship and the negatively coupled pathways. Similarly, in a local neural circuit where two input neurons connect to an output neuron, when the input synapse is inhibitory and the activation function is nonlinear, the correlation between input and output becomes distorted. This means that, in a linear temporal feature space, these closely connected neurons may appear far apart (Fig. 1e, see also Note S1).

An alternative measurement to describe the similarities of temporal features in neural dynamics is the shared information, rather than linear correlation. In the case of the logical gate, we calculated the mutual information (MI) between the input signal and output signal and found MI remained relatively high despite the non-linear signal processing (Fig. 1d). Back to neurons, although wired neurons could fire asynchronously, they share common hidden states. In a simple input–output circuit, given the input neuron activity, one should be able to predict the output neuron activity with some degree of certainty. A generalization of this intuition in large populations of neurons is that knowing a part of the neural population, the rest of the network should be statistically predictable, implying a lower-dimensional latent variable may describe the population activity [38–40]. In other words, if functional traces of neurons are regarded as a neural dynamics space, neurons belonging to a functional union should reside within an identical subspace spanned by neurons in that cluster (Fig. 1c). Identifying those subspaces from the whole neural space thus provides a route to discovering functional types from the mass of neurons.

We propose a framework for unsupervised discovery of subspaces from temporal features of neural activities. This is an instantiation of the anchor-guided subspace clustering algorithm [41–43]. Subspace clustering algorithm assumes that data points from the same cluster are drawn from one low-dimensional subspace. The main characterization and difficulty of subspace clustering is that points from the same subspace could be far from each other. Conversely, points from different subspaces could lie very closely (Fig. 1f). Therefore, accurate division of subspaces ensures the accuracy of clustering performance.

To accurately distinguish the subspaces formed by the neuron traces, we employed a two-step procedure: first, generating a similarity matrix $S$ that describes whether two points belong to the same subspace, and second, applying spectral cluster [41] on the undirected graph defined by *S* (Fig. 1g, see the section entitled ‘Methods’). It has been proved [44] that a similarity matrix *S* could be built by generating a sparse self-representation of each data point, $X \approx SX$, where $X$ is the concatenated neural activity matrix, $S$ is a sparse self-regression matrix (see the section entitled ‘Methods’). By finding the sparsest representation of the neuron, ${s}_{ij}$ describes whether neuron $i$ and neuron $j$ belong to the same subspace. To alleviate the computational load, the pipeline begins by identifying representative traces (called anchors $A$) from pre-processed neural activities and then regresses the neuron traces on the found anchors using a quadratic optimization algorithm with sparsity constraint. The regression coefficient of anchors forms the affinity matrix $Z$. The number of anchors and the sparsity regularization parameter balance the computational cost and the algorithm performance (Fig. S1a–c). The similarity matrix $S$ is then constructed based on the affinity matrix $Z$. Finally, spectral clustering is used to divide the neuron population into distinct groups. The aim of this clustering method is to minimize shared information between clusters, and obtain aggregated subspace clusters lying in low-dimensional latent spaces. We refer to the clustering result of this framework as the functionomics cluster of each neuron.

### Quantitative benchmarking within a simulated modularized dynamical system

**Figure 2.**
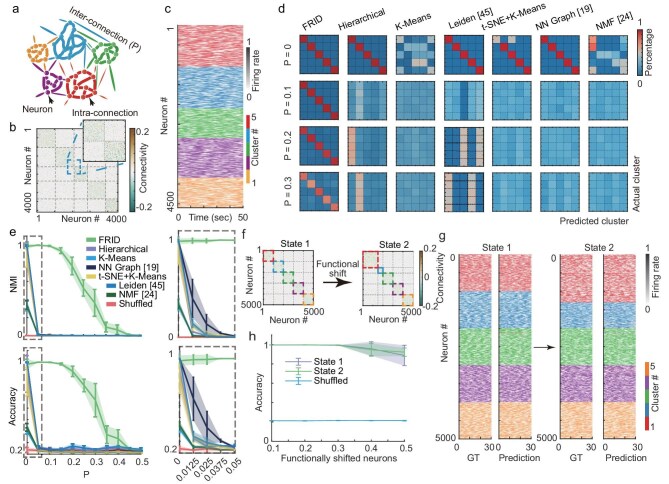
Evaluation of FRID in identifying functional clusters through numerical simulation of modularized dynamical systems. (a) Diagram of a complex dynamical system containing both intra- and inter-connections of functional clusters to mimic the functional neural network. The density of inter-cluster connection is denoted as P, which introduces crosstalk in different functional clusters. (b) Visualization of single-neuron-level functional connection matrix of the simulated system, which is the GT. (c) The spontaneous dynamics of the simulated system, different colors mark different functional modules. (d) Comparisons of the confusion matrices obtained by FRID, hierarchical clustering, K-means clustering and K-means clustering with AE learning, t-SNE with K-means method, NN graph [23], NMF with spectral clustering [28] under different inter-connection strength P. Column, *P* increasing from 0 to 0.3, row, different clustering methods. (e) Left, comparisons of the NMI and accuracy between inferred functional cluster results and the actual modules defined by simulation. Right, zoom-in illustration of NMI and accuracy changes among different methods for *P* ranging from 0 to 0.05. For each inter-cluster connection density *P*, we have *n* = 10 repeats using different randomized connectivity matrices, mean ± SD. (f) Illustration of the sudden change of connection matrix during state shifts. Half of the randomly selected neurons shift from Cluster 2 to Cluster 1, and the connectivity related to these neurons is rewired. (g) An example of the GT and predicted functional cluster before and after state shifts from State 1 to State 2 (30% neurons in Cluster 2 shift), illustrating that FRID can successfully identify the functional clusters accurately with high temporal resolution. This property is critical to analyze the changes of the functional roles of each neuron. (h) The accuracy of the inferred cluster with the increase of functionally migrated neuron number. We performed *n* = 5 repeats using three different randomized connectivity matrices. mean ± SD.

One of the challenges in studying functional circuit *in vivo* is the lack of GT [45]. To validate the effectiveness of our framework, we simulated a recurrently connected dynamical system with known GT (see the section entitled ‘Methods’). Neurons are divided into five modules. Within the module, neurons are connected through a sparse connection matrix. Across the modules, neurons were connected even more sparsely, controlled by a probability variable $P < 1$, which describes the proportion of non-zero connections between modules. (Fig. 2a and b) [17,46]. With randomly initialized connectivity drawn from a specific distribution [47,48], the simulated network enters a chaotic phase and generates spontaneous activity without external stimuli (Fig. 2c). We gradually increased the connectivity intensity between clusters, so that the degree of modularization organization of the network eventually deteriorated.

For comparison, we tested a wide range of clustering methods, including correlated-firing-based methods (K-means and hierarchical clustering), co-tuning-based methods (tensor component analysis [27]), spiking-model-based methods (Bayesian clustering [33] and Functional Encoding Units (FEUs) [35]), pairwise-similarity-based methods (N-nearest-neighbors (NNG) [23], NMF [28], Leiden [49] and Granger causality) and low-dimensional feature similarity-based methods (Uniform Manifold Approximation and Projection (UMAP), Independent Component Analysis, t-distributed Stochastic Neighbor Embedding (t-SNE) and auto-encoder (AE), followed by K-means methods). FRID significantly outperforms all these methods (Table S1 and Fig. S2). Notably, when the inter-module connectivity is non-zero, correlated-firing-based methods can only achieve chance-level accuracy (23%–25% when *P* = 0.05), while our proposed subspace-based method reaches near 100% and maintains a high accuracy of 98% even when *P* = 0.2 (Fig. 2d and e).

The number of clusters is a hyperparameter in FRID, which is unknown in empirical recordings. Fortunately, the singular values of the affinity matrix provide useful guidance while choosing the number of clusters (see the section entitled ‘Methods’). The number of large singular values suggests the number of hidden subspaces [41], which is validated by our simulation result (Fig. S3a). Moreover, through simulation, we found that FRID remains robust to the errors in estimating the cluster number. Even with incorrectly specified cluster numbers, FRID does not arbitrarily mix different functional clusters. If the assumed cluster number is smaller than the GT, FRID merges several hidden subspaces into one cluster. And when the assumed cluster number is larger, FRID splits some clusters into smaller subclusters. By merging these smaller subclusters, the accuracy could be largely recovered (99% in 1-more cluster case and 97% in 2-more cluster case, Fig. S3b and c).

Another important consideration is that functional circuits are known to be dynamically shaped by the external environment and internal state of the animal [31,46,50,51]. Consequently, the collective behavior of neural activities is non-repeatable and highly diverse across sessions, posing challenges for aligning the subspaces across sessions. Consistent functional assignment across sessions depends on subspace alignment between different feature spaces. The core assumption we made for alignment is that consistent functional connectivity underlies the seemingly unpredictable instantaneous activity of neurons within a functional module. In FRID, the temporal feature space of subspace clustering is self-constructed from the instantaneous population activity of a specific recording session, implying that the affinity matrix shall remain consistent if anchors are aligned. Therefore, we align multiple sessions via a temporally concatenated way of anchor generation, which guides the combination order of the feature generation so that the underlying signal sources remain aligned. The anchor-based regression coefficients are then concatenated across sessions for the cross-session affinity matrix (Fig. S1d, see the section entitled ‘Methods’). This enables the observation of temporal shifts in the functional roles of individual neurons.

We simulated the dynamic shift of the functional organization by rewiring the connectivity between clusters when *P* = 0.1 (Fig. 2f). A part of neurons in Cluster 2 are assumed to functionally drift to Cluster 1 at a certain time point, and the connectivity of these neurons is updated so that their connectivity follows a new distribution based on new module assignment (see the section entitled ‘Methods’ for detailed simulation parameters). With the multi-session clustering of FRID and alignment pipeline, we observed the state-dependent shifts of the functional clusters (Fig. 2g). Neurons could be correctly assigned to their dynamically shifted clusters, and the classification of non-shifted neurons is little influenced. When 35% of neurons in Cluster 2 shift, FRID achieves an average accuracy of 98.9% in Clusters 1 and 2 and an average accuracy of 99.7% in other clusters. As the degree of rewiring increases, the algorithm maintains robust with the global accuracy above 95% even when the system has up to 40% of the neurons in Cluster 2 shifted to Cluster 1 (Fig. 2h). Therefore, our results demonstrated that in the simulated network, the dynamically evolved functional clusters can still be resolved even with the existence of functional shifts.

### Functionomics beyond functional brain areas

We next turn to experimental data for further benchmarking of FRID against previous clustering methods. We aimed to determine whether the functional modules we identified are consistently preserved across different behavior protocols, and how these modules correspond to the coarse-grained anatomical functional brain areas.

**Figure 3.**
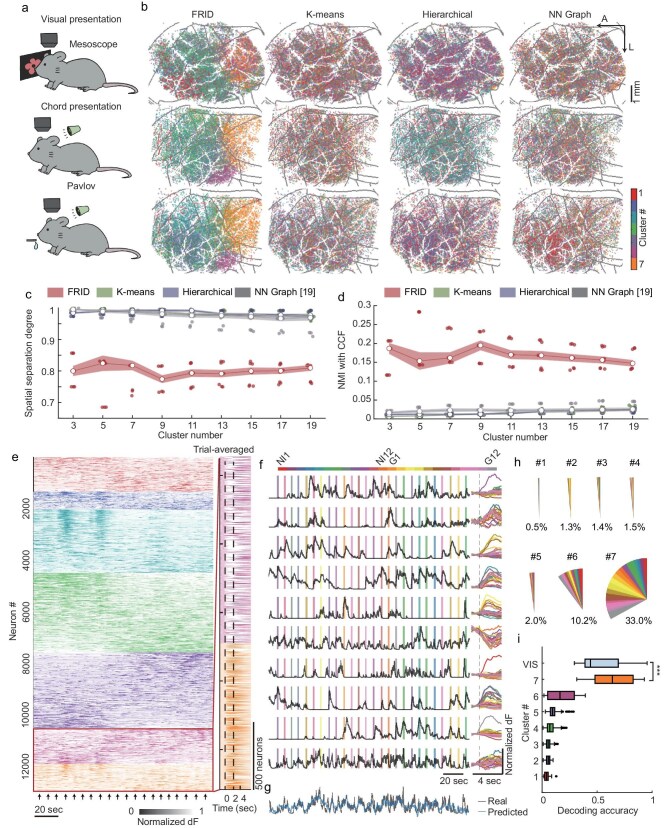
FRID enables stable unsupervised identification of distinct functional microcircuits in various behavioral protocols of head-fixed mice. (a) Schematic of three behavior protocols of head-fixed mice. Left, passive visual stimulus task with presentations of NIs and Gs. Middle, passive auditory stimulus task with presentations of different chords. Right, auditory cued Pavlov conditioning task. (b) Spatial distributions of functional clusters obtained by our FRID method, K-means clustering, hierarchical clustering and NN graph-based clustering during three different behavior protocols described in panel (a). (c) Plots of SSDs of functional clusters versus different clustering numbers. The dot represents the median, and each filled dot represents an individual run. We used three protocols and three runs for each protocol and a specific cluster number. (d) Same as panel (c), but showing the NMI between spatially defined CCF brain areas and identified functional clusters. (e) Temporal calcium traces of neurons recorded in visual presentation task from an example mouse. Neurons are colored based on their functional clusters. Each arrow at the bottom indicates a stimulus trial. The trial-averaged responses of Clusters 6 and 7 are shown on the right. (f) Ten example neurons from Cluster 7. Single-trial traces (left) and trial-averaged (right) traces are shown. Each shaded bar indicates a visual stimulus colored by content (12 natural images and 12 different oriented gratings). The same colormap is used in the trial-averaged curve. Neurons are highly heterogeneous within the same cluster. (g) The last neuron in panel (f) does not show any apparent visual response or selectivity but is still clustered into Cluster 7. Because it could be approximated by the linear combination of other neurons from Cluster 7, indicating they belong to the same subspace. Real and predicted (by 500 randomly sampled neurons from Cluster 7) traces of this example neuron are shown. (h) The proportion of image-selective neurons in each of the seven functional clusters. Image-selective neurons are massed into Clusters 6 and 7 in an unsupervised manner. (i) The decoding accuracy of neurons in each cluster, and the CCF-defined VIS. FRID identified neuron populations with significantly higher decoding accuracy than VIS, one-sided Wilcoxon rank sum test, *P* < 10^−18^ (4 mice, each with 5 technical repeats of FRID and 10 bootstrapped sampling of neurons). The whiskers show the maximum and minimum value of the distribution, the bound of the box shows the upper and lower quartiles, and the line in the middle shows the median.

Using transgenic mice expressing GCaMP6s in layer-2/3 cortex (Ai162^52^ × Rasgrf2-2A-dCre), we captured the calcium responses of around 10 000 neurons on the right hemisphere simultaneously with a RUSH3D [12] (Fig. S4a and Movie S1). We used both a passive sensory exposure (image and chord presentation) protocol and an active engagement protocol (Pavlov conditioning with a chord serving as the cue) (Fig. 3a) (2 mice). Neurons were registered to an oblique projection cortex atlas of Allen Mouse Common Coordinate Framework (CCF), using retinotopic mapping [53] from intrinsic signal imaging as the registration reference (Fig. S4b–e). The field of view (FOV) covers multiple functional brain areas including visual, auditory, motor, retrosplenial and somatosensory cortex (Fig. S4f and g).

It is well established that the cortex network is modularized, with specialized functional areas that each contribute to specific cognitive functions [2]. CCF atlas offers a widely used standardized functional area division standard in mice. Thus, the agreement between the functional cluster and the CCF brain area can be regarded as a course validation benchmark to evaluate the algorithms. Guided by the singular value spectrum (Fig. S6a), we first clustered neurons into seven clusters by our proposed FRID framework and other clustering methods (Fig. 3b). The functional clusters are then mapped onto the cortex atlas. Notably, among all methods, FRID automatically organized neurons into spatially more recognizable patches on the atlas without any spatial footprint as prior guidance (Fig. 3c and Fig. S5).

Using the peri-stimulus time points (Fig. S8b), instead of tuning any parameters (Fig. S2e and f), slightly improved the performance of K-means. By constraining the used time points in peri-stimulus period, K-means could roughly identify the responsive neuron population, but failed to cluster non-responsive neurons. In comparison, FRID is an unsupervised data-driven method that learns the sparse representation of one neuron using all the representative anchors generated from the whole population. Therefore, its clustering performance does not rely on specific external stimuli-triggered activity, but largely on the internal dynamics of neurons, thus could be used on purely spontaneous activities of cortical neurons (Fig. S8c). This demonstrated the data-driven discovery capacity of FRID framework without prior knowledge and supervised labeling. We then systematically varied the number of clusters, FRID reveals a gradually fine-grained functional atlas (Fig. 3b and c). We calculated the normalized mutual information (NMI) between the CCF brain areas and the functional clusters (Fig. 3d). Our proposed framework achieves a significantly higher NMI with CCF than other methods (one-sided Wilcoxon rank sum test, $p = 1.95 \times {10}^{ - 3}$ in all cluster numbers, three datasets, algorithmically repeated three times each). In addition, the average spatial separation degree (SSD) of FRID is around 0.78, indicating spatial organization of the functional areas, while K-means, hierarchical clustering, and N-nearest graph are around 0.99 without identifying the spatial distributions.

The representative tuning properties of each functional cluster are visualized (Figs S7–S11). In the visual presentation protocol, neurons from Clusters 6 and 7 show distinct tuning responses to the natural image (NI) and grating (G) patterns. These neurons are mostly (94.5% of Cluster 6, 94.6% of Cluster 7) distributed in visual areas (Fig. S13a). We found FRID grouped 76.1% selective neurons into Clusters 6 and 7 in an unsupervised manner, indicating the functional role of these visual-coding clusters (Fig. 3h, Fig. S13b and c). And 53% neurons from Cluster 7 and 23.5% neurons from Cluster 6 respond to the onset and the offset of the visual stimuli, respectively (Fig. 3e), suggesting complementary neuron populations [54]. Neurons from Cluster 7 show highly heterogeneous single-trial responses as well as trial-averaged responses (Fig. 3f and Fig. S14d). Notably, some neurons do not show any apparent visual response or selectivity but still lands in Cluster 7, because its activity could be predicted from other neurons in the same cluster, indicating they share a common subspace (Fig. 3g). This highlights the key distinction between FRID and other correlated-firing-based clustering methods.

We used a unique principal component analysis (PCA)-based analysis (see the section entitled ‘Methods’) to illustrate the most representative visual responses of neurons. The result shows that the first three unique principal components (uPCs) have little remaining component after diminishing the sharing components, which is dominated by body movements (Fig. S13d and e). We noticed uPC 4 from Cluster 7 distinctly separated G stimulus from NI stimulus (Fig. S14a). This suggests that in the visual population (Cluster 7), the primary coding dimension distinguishes natural imaging from the G pattern, and a minority of coding capacity is used to distinguish between different G orientations and NI identity. Neither the other visual-cortex-landed cluster (Cluster 6) nor the anatomically defined population (visual cortex (VIS)) shows such property (Fig. S14b and c). Similarly, in auditory passive exposure protocol. Chord selective neurons are predominantly localized to Cluster 6, partially overlapping with the auditory cortex and surrounding areas (Fig. S14e–g). To facilitate the use of FRID among various protocols and datasets, a graphic user interface is developed that integrates the entire pipeline of data loading, parameter tuning, clustering and result visualization (Fig. S15).

FRID identifies functional clusters that are consistent but not identical to CCF. To evaluate their difference, we performed a decoding experiment with visual stimulus protocol. We found that visual-information decoding ability of Cluster 7 neurons is significantly higher than CCF-defined VIS neurons (4 mice, each with 5 technical repeats of FRID and 10 bootstrapped sampling of neurons, *P* < 10^−18^, Fig. 3i and Fig. S13f). This indicates that although the identified clusters correspond to CCF, they are more task-informative than anatomically defined brain areas, revealing additional information on cell type identity beyond anatomy.

Beyond our demonstration of FRID on layer 2/3 cortex activity of mice, the framework generalizes well across species and behavioral protocols. We tested the framework on additional datasets, including calcium imaging of zebrafish whole-brain activity under visual stimuli [55] and whole-brain imaging of *Drosophila* under odor stimuli [56]. Spatially well-organized functional clusters are identified in both zebrafish and *Drosophila* (Fig. S16a and b). Moreover, we tested the method on a pseudo neural population [29] constructed from mice primary VIS obtained by two-photon microscopy during visual stimulation. The pseudo population is a concatenated result of 17 sessions from 4 mice. Interestingly, FRID distinguished neurons recorded in the same session (Fig. S16c) in an unsupervised manner. This indicates internal dynamics primarily shape neural network activity, whereas external task-driven modulations play a comparatively minor role.

To further examine the applicability of FRID beyond calcium imaging, we performed a proof-of-concept analysis using one publicly available Allen Brain Observatory Neuropixels recording session [57]. Spike trains recorded during repeated natural-movie presentations were converted into continuous firing-rate representations before FRID analysis. FRID organized the recorded units into eight clusters with distinguishable temporal activity patterns (Fig. S16d and e). The identified clusters showed non-uniform anatomical compositions across visual cortical, hippocampal and subcortical regions, although anatomical labels were not supplied to the algorithm (Fig. S16f). These results provide a proof-of-concept demonstration that FRID can be applied to large-scale electrophysiological recordings after appropriate firing-rate estimation.

### FRID demixes dynamic sensory-coding communities without supervision

**Figure 4.**
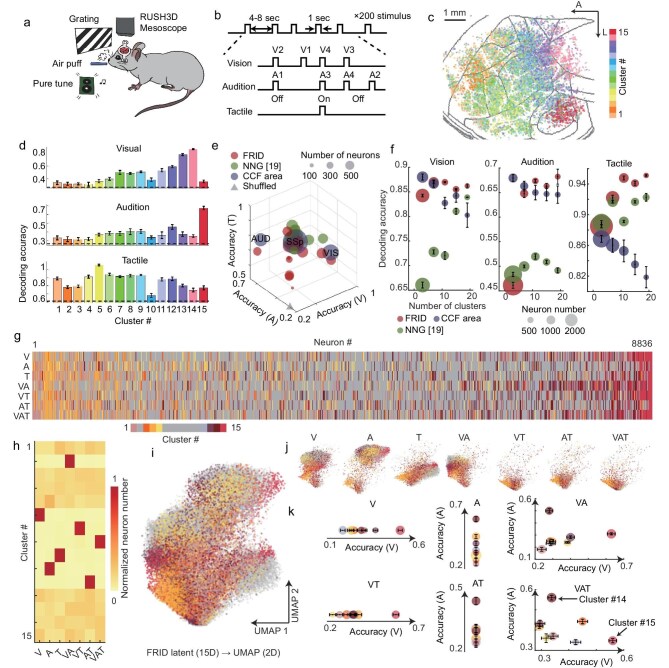
Demix of sensory information in a multi-sensory presentation protocol and the dynamics of functional architecture. (a) Schematic of multi-sensory presentation protocol for head-fixed mice. (b) Three types of sensory are presented either simultaneously or respectively. The contents of visual and auditory stimulus are randomly generated from four possible alternatives. The air puff is either on or off. (c) Spatial distribution of 15 functional types. (d) Decoding accuracies of the best-coding cluster among all functional clusters with the increase of fineness of division. Results of two methods and CCF among three sensory modalities are shown, respectively. Error bars show SEM of 10 bootstrapped sampling of neurons from that cluster. mean ± SEM. (e) The decoding accuracies of visual (V), auditory (A) and tactile (T) sensory information of each functional cluster displayed in 3D. We compared the results obtained by FRID, NNG, CCF brain area and shuffled data, marked by different colors. Each dot represents a cluster, and the size of the dot represents the number of neurons in the cluster. (f) Decoding accuracies of the best-coding cluster among all functional clusters with the increase of fineness of division. Results of three methods and three sensory modalities are shown, respectively. Error bars show SEM of 10 bootstrapped sampling of neurons from that cluster. (g) Shift of the functional roles of neurons that belong to conservative functional clusters across sessions indicated by the functional. Trials are divided into different sessions, such as visual-only trials (V), auditory-only trials (A), simultaneous visual and auditory trials (VA) and simultaneous visual, auditory and tactile (VAT) trials. Neurons are sorted according to the averaged cluster index in seven session types. (h) Number of neurons in each cluster of each session. Normalization is conducted in each row, so that we show the fraction of neurons in different sessions. (i) The visualization of cross-session aligned latent space (15D) of FRID using UMAP (2D). (j) Latent space of each session. (k) Decoding accuracy of different sensory modalities in each session. Each cluster is represented as a filled dot in the decoding space. The size of the dot represents the number of neurons in that cluster. Error bars show SEM of 10 bootstrapped sampling of neurons from that cluster. We show that distinct sensory information is held by same functional clusters containing dynamically recruited neurons.

One of the desirable attributes of functional module clustering is the ability to disentangle behavioral variables through cluster separation. In an ideal modularized system, specialized modules should be responsible for specific tasks and remain unrelated to others. To test whether FRID could effectively demix sensory information in the cortex, we conducted a multi-sensory stimulation experiment on head-fixed mice. Three different sensory modalities were presented to the head-fixed mice (Fig. 4a and b). Visual information consisted of four different oriented G bars, auditory information was delivered as four different frequency pure tunes and tactile information was provided as an air puff targeted at the face and whisker area. Different sensory contents were combined and delivered in a pseudo-random order, and in some trials, one or two modality inputs were randomly omitted. Neurons were grouped into 15 distinct functional types using FRID (Fig. 4c). Again, most functional clusters show roughly aligned but non-identical spatial patterns with known CCF brain areas. We then used these clusters to decode the presented stimuli, to examine whether the sensory information was represented by specific functional clusters. A k-nearest neighbor (NN) classifier based on the population vector was applied to evaluate the decoding fidelity (Fig. 4d). The decoding accuracy indicated that most visual information was preserved in Clusters 13 and 14, and auditory information in Cluster 15. A large proportion of neurons in these clusters were localized within the primary visual area and auditory area (Fig. S17), consistent with well-known primary sensory perception hubs [57–59]. In contrary, tactile information was more diffusedly distributed across clusters. The cluster with the highest decoding accuracy was Cluster 5, which was roughly corresponded to the somatosensory cortex, including the barrel, nose and mouth field.

We then investigated how these clusters distribute in a decoding space defined by the decoding accuracy across the three sensory modalities (Fig. 4e). We found that functional clusters identified by FRID spanned a broader range in the decoding space compared with those identified by other methods. Several small clusters with high decoding accuracy emerged, indicating that FRID automatically identified stimuli-related neurons with a high decoding signal-to-noise ratio [60] in an unsupervised manner. To assess how the number of clusters affects the ability to demix task-related neurons, we systematically increased the cluster number in our algorithm. As the number of clusters increased, smaller and more refined clusters were revealed (Fig. 4f). The decoding accuracy of FRID-identified clusters remained relatively higher than that of clusters obtained by other clustering methods. When the cluster number was large, FRID successfully identified the most informative neurons in VIS or auditory (AUD) cortex without supervision, achieving even higher decoding accuracy than randomly sampled neurons from VIS and AUD. This demonstrates FRID’s capacity to extract the key coding component in an unsupervised manner.

To understand how functional architecture changes when external stimulation shifts, we applied the multi-session FRID clustering pipeline to the multi-sensory stimulation data. Trials were algorithmically realigned into separate states based on the presented sensory modality. These states included single-modality input trials, such as visual-only (V), auditory-only (A) and tactile-only (T) trials, as well as multi-sensory input trials, such as visual and auditory (VA) states. Functional modules with consistent internal structure were assigned to the same functional cluster across different states. We found that neurons underwent functional reassignments across states (Fig. 4g and h and Fig. S18a). For each neuron, we first identify the cluster it appeared most frequently, and then quantified the number of states in which it appeared in that preferred cluster (Fig. S18f). On average, 74% ± 8% (mean ± SEM, *n* = 3 mice) of neurons appear in the preferred cluster for <4 states, and only 17% ± 6% of neurons remained in their preferred cluster for >4 states. The cluster organization was more stable than the shuffled case (Fig. S18f), and subdividing trials within the same state revealed higher intra-state than inter-state similarity (Fig. S18c and d), excluding the dynamics from pure across-trial variability.

The latent space obtained by FRID also shifted under different combinations of input modality, suggesting state-dependent changes in functional architecture (Fig. 4i and j). While tactile input was present, neurons clustered toward a distinct region of the latent space, and similarity analysis revealed greater functional resemblance among these states (Fig. S18b). These findings suggest that when tactile is combined with vision or audition, it elicits a widespread cortical response, leading to similar large-scale brain states. Despite such pronounced dynamics, the decoding analysis showed that sensory information remained preserved within the consistent functional clusters identified by FRID (Fig. 4k). Visual information was largely preserved by Cluster 15 and auditory information by Cluster 14. This validates the functional stability of our identified functional modules despite the dynamic reorganization of functional circuits.

In multi-sensory integration sessions, we noticed the dynamic recruitment and release of neurons between sensory hubs, suggesting the functional roles of sensory neurons may shift in the sensory integration process [61]. Tuning curves of example neurons (Fig. S19a and b) revealed that even when the preferred sensory modality was absent, neurons could still be correctly assigned to the corresponding functional cluster. In addition, some neurons exhibited non-linear integration effects when multiple sensory inputs were presented simultaneously. These results demonstrate that FRID can identify unified functional clusters across divers task states and reveal the dynamic reconfigurations of cortical functional circuits.

### Identification of reorganization of functional architectures during ischemic stroke

Brain disorders such as focal ischemic stroke can also alter the functional architecture of the cortical network [62–64]. However, due to the high individual variability of stroke lesions, the complex responsive properties of functional neurons and the disruption of the anatomical distribution of function, cellular-level functional plasticity of these networks remains poorly understood.

**Figure 5.**
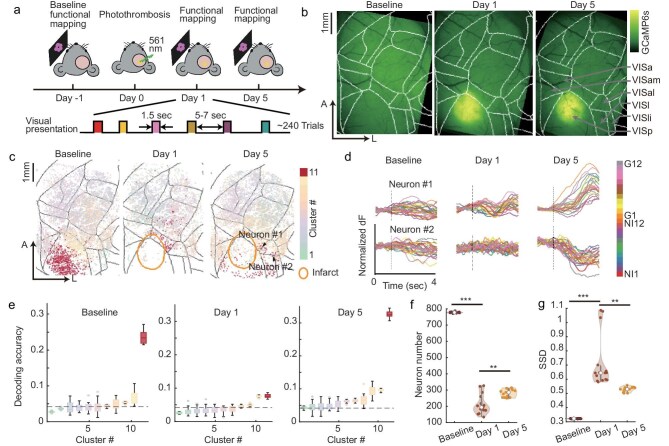
FRID reveals reorganization of functional atlas at single-cell resolution during ischemic strokes. (a) Diagram of the functional-mapping protocol for the ischemic stroke model induced by photothrombosis. Visual stimulus contains 24 different images including both static grating patterns and natural images. (b) Center view of single-frame light field image captured before photothrombosis (baseline), 1 day and 5 days after photothrombosis. The infarct could be discerned as a bright area. (c) Spatial distributions of the functional atlas identified by FRID on the day before photothrombosis (baseline), 1 day and 5 days after photothrombosis. The area of infarct of the ischemic stroke is indicated by the solid line. Clusters are colored based on their decoding accuracy to the visual stimuli. (d) Response curves of two example neurons (N #1 and N #2) to 24 different images. These neurons exhibit remapped response properties during recovery from stroke. The location of these neurons is indicated by arrows in panel (b). (e) The populational decoding accuracy of different functional clusters in three different days. Decoding ability of visual-related cluster (#11) decreases on day 1 and recovers on day 5. Shuffled decoding result is shown by the dashed line. The boxes show 20 independent bootstrap samples of a part of the neurons from each cluster. The whiskers show the maximum and minimum value of the distribution, the bound of the box shows the upper and lower quartiles, and the line in the middle shows the median. (f) The number of neurons in the same visual-related cluster (#11) on three different days. Each filled dot represents an individual run of FRID, the white dot shows the median. Total 15 technical repeats are performed for each day. (g) The SSD of Cluster #11 in three different days. The elements of the violin plot share the same meaning as in panel (e). **, *P* < 0.01, ***, *P* < 0.001, two-sided Wilcoxon rank sum test.

We combined FRID with a photothrombosis model to investigate the disruption and recovery of specific functional clusters. Photothrombosis was induced by rose Bengal and 561-nm laser exposure targeted to the VIS in three mice. A visual presentation protocol was conducted before and after photothrombosis (Fig. 5a and b). We used calcium responses collected during visual exposure for FRID analysis (Fig. 5c and Fig. S20). During the early phase of stroke (Day 1 after photothrombosis), visual function was extensively impaired in two mice with larger infarcts. Both the number of neurons in the visual cluster and the decoding performance of this cluster decreased relative to baseline (Fig. 5e and f). However, in the functional atlas of Day 5, we observed functional recovery of some neurons near the infarct area. The decoding ability of the visual-related functional cluster also improved (Fig. 5e).

As previously demonstrated, the neurons identified by FRID can exhibit heterogeneity in their tuning properties (Fig. 3f). Therefore, 48% of neurons in Day 5 Cluster 11 are significantly activated by visual stimulus, 32% neurons are deactivated by visual stimulus and the rest 20% neurons do not show apparent tuning. This highlights FRID’s distinction from supervised or correlated-firing-based methods. Notably, the spatial distribution of the visual-coding community covered a broader range of anatomical areas on Day 5 compared with baseline (Fig. 5g and Fig. S20). For instance, some neurons from higher-order VIS were newly included in the cluster. Some of these neurons exhibited selective responses to visual stimuli that were absent before the stroke (Fig. 5d). The anatomical functional areas these neurons occupied were mostly lateral visual areas (VISl, VISli and VISal), rather than non-visual cortical areas. Therefore, our results support the occurrence of functional remapping during post-stroke recovery; however, the extent of this plastic functional remap may be restricted by anatomical and hierarchical factor [62].

### FRID reveals modularized coding in decision making

While some sensory information is believed to be coded by specific functional areas, other task variables, such as choice [50], action [20,21] and engagement [65,66] are known to trigger widespread activity across the brain. Based on FRID results, we asked how these different task variables could be decoded from identified functional clusters.

**Figure 6.**
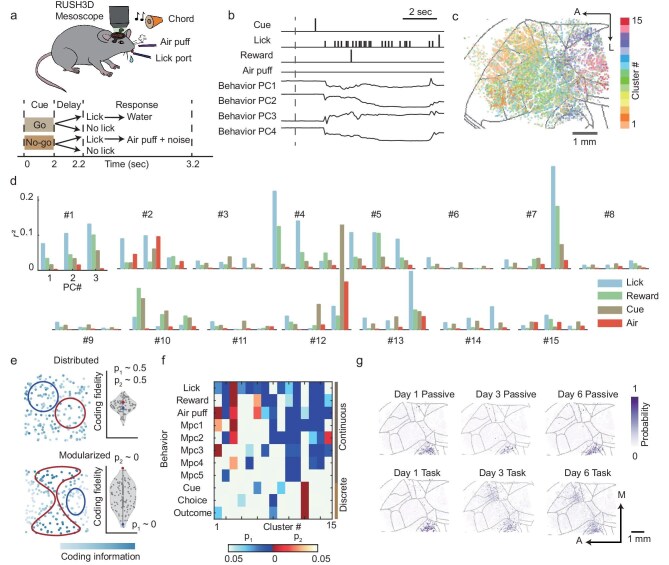
Functional atlas in Go/No-Go task shows widespread motion-driven activity and modularized coding properties of task variables. (a) Schematic of the Go/No-Go protocol with head-fixed mice. The mice earn a reward by licking at the presence of the correct chord, and avoid the punishment by holding licking in the alternative case. (b) The change of task-related variables and spontaneous variables in an example trial. (c) Spatial distribution of functional clusters. (d) Explained variance of the PCs of each cluster by the behavior variables. A linear model is used to fit the PCs with behavior event kernels. (e) Diagram of distributed and modularized coding strategy. By utilizing shuffling tests, we can show whether the behavior information is specifically coded by the modularized community. p1 and p2 are used to indicate the percentile of the empirical data, p1 = 1 − p2. (f) *P* value of the encoding fidelity of functional clusters. *P* = min (p1, p2). (g) The spatial distribution of the cue-best-coding cluster in the active engagement and passive exposure sessions on the 1st, 3rd and 6th training day. The training process dynamically reshaped the functional arrangement.

To answer this question, we trained head-fixed mice to perform an auditory-cued Go/No-Go task. The mice earned a reward by licking at the Go trial, and avoided an air puff by withdrawing its licking at the No-Go trial (Fig. 6a and Fig. S21b). Licking behavior and body movements were recorded by photodiodes around the jaw and an infrared camera targeting the face and body (Fig. S21a). Various task variables were extracted, including cue identity, licking time points, reward delivery, air puff delivery and the principal components (PCs) of the body movements (Fig. 6b). Fifteen functional clusters were identified by FRID (Fig. 6c and Figs S22 and S23). As previously reported [20,21], even in disentangled functional clusters, the first PC of each cluster was largely explained by the spontaneous body movements and showed high correlation (Fig. S21d, see the section entitled ‘Methods’). This suggests a global driving influence of spontaneous behaviors across all neuron populations. However, the second and third PCs of some neuron clusters were less explained by body movements, suggesting the presence of other independent latent driving forces, some of which are task related. For instance, licking behavior drives the third PC in Cluster 7, and cue presentation drives the third PC in Cluster 12 (Fig. 6d).

One way of testing distributed versus modularized coding strategy is by sampling neurons and observing the distributions of coding fidelity (Fig. 6e, see the section entitled ‘Methods’). Under the extremely distributed encoding assumption, information is evenly shared by neurons. Therefore, random sampling from the neural population results in similar decoding performance across task variables. Conversely, if a specialized module is responsible for the coding, while other neurons are unrelated, the sampling strategy strongly influences decoding performance. Sampling from a high task-related population leads to a significantly higher decoding performance, whereas sampling form unrelated neurons yields significantly lower performance. We tested our identified functional clusters’ decoding performance and compared them with randomly sampled neuron clusters with the same neuron number (Fig. 6f and Fig. S21e and f). We calculated the percentile of the empirical data decoding accuracy within the shuffled distribution in the descending and ascending order as p_1_ and p_2_. Small values of p_1_ indicated significantly better encoding than randomly sampled neurons. We found most task variables have one or more specific coding functional clusters, including the first three PCs of body movements. This suggests that a modularization coding property holds in most of the behavior variables. Although body movements influence global neural activity, the proportion of shared information in movement behavior varies among functional clusters (Fig. S21g). Thereby, FRID could distinguish modules despite this global influence of movement.

Clusters 3 and 12 show significantly high decoding ability of task-related continuous variables and discrete variables. Cluster 3 contains distributed neurons mostly from the somatosensory area upper limb, lower limb and trunk; whereas Cluster 12 contains neurons primarily from the auditory cortex (Fig. S21h). An underlying circuit mechanism of conditioning is the broadcast of sensory stimulus information from the sensory cortex to the motor and somatosensory cortex [50,67]. From the perspective of functional circuit, we asked if this circuit is modulated by cross-day learning progress. Therefore, we performed functional clustering on successive training days. The mice showed extensive task learning over 6 days of training (Fig. S21c). The cluster with the highest cue-decoding accuracy during the cue presentation phase was considered the cue-coding cluster. We observed a gradually spatially expanding cluster with the ongoing training, suggesting a possible formation of a functional circuit linking the sensory cortex and the somatosensory cortex (*n* = 3 mice; Fig. S21i). These results suggest that the conditional cue triggers the learned licking behavior. Altogether, the cortex functionomics clustering result depicts a dynamically arranged neural circuit that could be shaped by long-term learning.

## DISCUSSION

We present an algorithmic approach to combine with mesoscale single-neuron resolution recording for revealing functional modules associated with specific tasks and subjects. Unlike widely used Pearson correlation or connectome-based methods, we propose using subspace identification as the metric for recognizing the functional microcircuits. The fidelity of the framework is demonstrated via its performance on a simulated dynamical system and its consistency with coarse-grained functional brain areas. We also demonstrate how coding information can be automatically demixed by FRID, suggesting its applicability to data-driven discoveries. Cross-session clustering provides a chance to link brain states with functional architecture shifts, which could potentially serve as biomarkers to distinguish different internal states of animals. Moreover, the long-term learning or development of the animal also reshapes the organization of functional architecture, which our method could be particularly well-positioned to capture.

For the unsupervised algorithm, the subspace is automatically identified by sparse representation. Therefore, the number of independent sampled time points plays a crucial role in the performance of the algorithm. In our presented cases, calcium responses recorded at 10 Hz over 15–30 min are used as the training dataset. The self-correlation of the calcium signal is relatively high due to the temporal dynamics limitations of GCaMP6s. In other recording modalities, such as voltage indicator imaging and electrophysiology, there is greater independence between sampling points. In these high-temporal resolution recording techniques, there may be potential to estimate functional modules from a shorter recording period, which facilitates the study of short-term functional shifts. The number of clusters is a hyperparameter determined by the user, depending on the desired division fineness. We show that FRID remains robust to different cluster numbers in a simulated network (Fig. S3). The singular values of the affinity matrix provide evidence of the underlying subspace number. In empirical data, the singular value spectrum is smoother than in the simulated case, likely because the cortex network is much more complex and inter-cluster connected, exhibiting less well-defined subspace division [41] (Fig. S3d). Choosing the cluster number becomes more empirical in these cases. We anticipate future work could incorporate hyperparameter choosing in the pipeline or address this problem in a hierarchical clustering approach. Additionally, other hyperparameters may influence clustering performance, such as the sparsity regularization term and the anchor number (Fig. S1a and b). A balance must be struck between performance, computational load and robustness. Moreover, sparse representation is only one of the many approaches to realize subspace clustering. Applying data-driven deep learning methods may further facilitate the discovery of non-linear information transfer between functional circuits, shedding new light on the cellular functional atlas studies.

Generally, FRID produces consistent functional clustering results across different individuals if the FOV is similar (Fig. S3d and e). But the result may be influenced by the neuron number in different functional clusters (Fig. S3f and g). For instance, if the number of neurons in a specific cluster is extensively high, these neurons may be divided into two separate subclusters due to global optimization.

Our proposed framework is a data-driven method suitable for discovery-aimed studies. We demonstrated that some well-known functional modules, such as visual, auditory and somatosensory cortex are identified in an unsupervised manner. There are also accumulating evidence that neurons self-organize into higher-order functional assemblies defined by shared dynamics, such as the motor cortex ‘information clusters’ [68], hippocampal place cell assemblies [69] and habenular ‘spontaneously organized activity clusters’ [70]. However, some clusters emerge from the algorithm whose function remains unclear. These mysterious clusters preserve information distinct from known clusters, yet their behavioral counterparts remain undiscovered. The number of neurons in these clusters is often considerably large. Some of these clusters are found related to arousal state (Fig. S24). Others may encode some more abstract information, such as memory [51,71], attention [72–74] or change-of-mind [75] during decision making. It is important to design new behavior protocols to determine the precise function of these clusters for fully understanding of the brain organization. Additionally, well-established causal relationship can only be determined by modulating specific functional modules. We anticipate these experiments to be carried out using optogenetic tools or lesion studies to validate function module delineation.

FRID is related to, but methodologically distinct from, latent-variable dynamical models such as Gaussian Process Factor Analysis (GPFA) [76] and Latent Factor Analysis via Dynamical Systems (LFADS) [77]. GPFA and LFADS primarily infer continuous low-dimensional trajectories that describe the temporal evolution of neural population activity, whereas FRID directly partitions individual neurons according to similarities in their intrinsic dynamical representations. Latent-variable models and FRID address complementary questions: the former characterize population-level temporal dynamics, while FRID characterizes the modular organization of neurons within potentially distinct dynamical subspaces. Future work could combine these approaches by applying neuron-level subspace clustering to representations learned by non-linear latent dynamical models.

Genomics and transcriptomics have already achieved extensive progress in modeling brain networks and treating brain diseases in a data-driven way. However, the intelligence of the brain essentially arises from the dynamic processes that can only be captured by *in vivo* functional recording. With the aid of recording techniques and data science, we believe functionomics provides a practical approach to systematically establish the functional map of the brain. Furthermore, integrating high-throughput transcriptome with *in vivo* large-scale recording could offer a systematic view of the genetic-functional pathway guiding neuronal development and activity.

## METHODS

### Anchor-guided large-scale subspace clustering

We use subspace clustering to study neuronal groups and their representative functions. Subspace clustering models each neural trace as a linear combination of other traces and optimizes the reconstruction loss to obtain the combination coefficients. The coefficients are interpreted as the similarity between corresponding neural firing rates.

Mathematically speaking, given a pre-processed neural trace matrix data $X$, the size of $X$ is $N \times T$, where $N$ is the number of neurons and $T$is the number of temporal sampling points. Subspace clustering aims to optimize the following problem:


\begin{eqnarray*}
\mathop {\min }\limits_S \left\| {X - SX} \right\|_F^2 + \alpha f(S)\quad {\mathrm{ s}}{\mathrm{.t}}{\mathrm{.}} - 1 \le {s}_{i,j} \le 1,
\end{eqnarray*}

where $f$ is a normalization function for regularization and $\alpha $ is the penalty factor. $S$ is commonly referred to as the similarity graph matrix. The solution can be obtained via a quadratic programming.

Given a large number of neurons as input, $S$ becomes a very large matrix, making direct optimization computationally and memory-intensive. To address this, a low-rank matrix approximation method is incorporated to mitigate this problem.

It is convenient to use anchor graph to construct a large-scale graph matrix. The key idea is to construct $X$ with representative anchors $A$ generated from $X$, reducing the size of $S$ to decrease the computational complexity while maintaining the clustering performance at the same time. Thus, the problem turns into an alternative form


\begin{eqnarray*}
\mathop {\min }\limits_Z \| {X - ZA} \|_F^2 + \alpha f(Z)\quad {\mathrm{ s}}{\mathrm{.t}}{\mathrm{.}} - {\mathrm{1}} \le {z}_{i,j} \le 1,
\end{eqnarray*}

where $A$ has a shape of $M \times T$, where $M$ is the number of anchors and $M \ll N$, selected using lite K-means clustering. $Z$ is the affinity matrix, and here the normalization function $f$ is chosen to be square of Frobenius norm, meaning $f(Z) = \sum\nolimits_i^m {\sum\nolimits_j^n {{{| {{z}_{ij}} |}}^2} } $. In this condition, $Z$ can be solved much more efficiently.

After $Z$ is obtained, the similarity graph matrix $S$ can be efficiently computed as $S = | Z |\centerdot {| Z |}^{\mathrm{T}}$ with the absolute value of the affinity matrix, since we consider both very positive and very negative weights as a strong connection.

We then leverage spectral clustering on $S$ to get the final results. Specifically, singular value decomposition is first applied to $| Z |$, meaning $| Z | = U\sum {V}^T$, where $U$ and $V$ are unitary matrices, representing the intrinsic feature of $| Z |$, and $\sum $ is the singular value matrix. The number of large singular values in $\sum $ suggests the number of hidden subspaces. Finally, K-means clustering is incorporated to first $k$ columns of $U$ to get the final clustering results.

### Multi-session subspace clustering

When dealing with multi-session data $\{ {{X}_1,{X}_2...{X}_K} \}\ $, where each ${X}_i$ contains ${T}_i$ time points, $i \in \{ 1,2...K\} $, $K$ is the session number. We first concatenate all the session along the temporal axis and send them to the lite K-means algorithm for anchor selection. After that, we split the anchors along the time axis back into the corresponding sessions and use quadratic programming to compute each ${Z}_i$, then we concatenate all the ${Z}_i,i = 1,2...K$ along the neuronal axis to get the whole matrix $Z$ that has the dimension $NK \times M$. With $Z$, we can calculate the similarity graph matrix $S = | Z |\centerdot {| Z |}^{\mathrm{T}}$, and apply spectral clustering on it to obtain the final cluster assignments. After clustering, we distribute the cluster identities across sessions. Neurons may get different identities in different sessions, which means that their functions may drift, however, the overall functional clusters are aligned across sessions.

### Animals

All experiments involving living animals were conducted in accordance with Tsinghua University guidelines and approved by the Institutional Animal Care and Use Committee at Tsinghua University, Beijing, China.

## Supplementary Material

nwag420_Supplemental_Files

## Data Availability

A demonstration of FRID framework could be found at https://github.com/Cai-yy/FRID. Simulation datasets and GUI codes are all available at GitHub. The calcium dataset is available from the corresponding authors.
