# Supplementary material for: Constructing mesoscale functionomics by neural dynamics subspace clustering
Source: Natl Sci Rev. 2026 Jul 11;13(15):nwag420. doi: 10.1093/nsr/nwag420 (PMC13463643; doi:10.1093/nsr/nwag420)
Supplement: Supplementary file 1 [file nwag420_supplemental_files.zip › Supplementary Information v17.docx]

Supplementary Information

**Constructing Mesoscale Cellular Functional Atlas by Neural Dynamics Subspace Clustering**

Yeyi Cai^†^, Xinhong Xu^†^, Guihua Xiao, Huaming Ling, Zuoqiang Shi, Minghuan Wang, Mingrui Wang, Fenghua Chen, Jiamin Wu^*^, Qionghai Dai^*^

**I. Supplementary Methods**

**II. Supplementary Note**

| **Note S1** | Toy models to explain the principle and insight of FRID |
| --- | --- |

**III. Supplementary Table**

| **Table S1** | Comparison of key insight, computational load, and accuracy on simulation datasets for different methods |
| --- | --- |

**IV. Extended Data Figures**

| **Extended Data Figure 1** | Hyperparameter evaluation and cross-session subspace clustering pipeline |
| --- | --- |
| **Extended Data Figure 2** | Evaluation of competing methods in identifying functional clusters through numerical simulation of modularized dynamical systems. |
| **Extended Data Figure 3** | Choice of the number of clusters and individual consistency. |
| **Extended Data Figure 4** | Large scale recording with real-time, ultra-large-scale, high-resolution 3D mesoscope (RUSH3D) |
| **Extended Data Figure 5** | Evaluation of different methods on experimental datasets collected under 3 different protocols |
| **Extended Data Figure 6** | The cluster number in empirical data |
| **Extended Data Figure 7** | Spatial temporal functional map in visual presentation task. |
| **Extended Data Figure 8** | uPC visualization of each cluster under visual presentation and the clustering result under different circumstances. |
| **Extended Data Figure 9** | Spatial temporal functional map in chord presentation task. |
| **Extended Data Figure 10** | uPC visualization of each cluster under chord presentation. |
| **Extended Data Figure 11** | Spatial temporal functional map in Pavlov protocol. |
| **Extended Data Figure 12** | uPC visualization of each cluster under Pavlov protocol. |
| **Extended Data Figure 13** | Analysis on the clustering result of visual presentation protocol. |
| **Extended Data Figure 14** | Example neurons from the visual presentations task and the result of auditory presentation task. |
| **Extended Data Figure 15** | Screenshot of the graphical user interface (GUI) of FRID. |
| **Extended Data Figure 16** | Evaluation of the framework on other datasets. |
| **Extended Data Figure 17** | Spatial distribution of functional clusters in multi-sensory task. |
| **Extended Data Figure 18** | Analysis of the clustering result in multi-sensory presentation protocol. |
| **Extended Data Figure 19** | Functional shift in multi-sensory presentation protocol. |
| **Extended Data Figure 20** | Dynamics of functional cluster during ischemic strokes. |
| **Extended Data Figure 21** | Go/No-Go protocol |
| **Extended Data Figure 22** | Spatial temporal functional map in Go/No-Go protocol. |
| **Extended Data Figure 23** | uPC visualization of each cluster under Go / No-go protocol. |
| **Extended Data Figure 24** | Spatial distributed cluster and its relationship to pupil size |

**V. Supplementary Videos**

| **Video S1** | Mesoscale neural recording with real-time, ultra-large-scale, high-resolution 3D mesoscope (RUSH3D) |
| --- | --- |

# Supplementary Methods

**Visualization of the latent space of FRID**

For visualizing the latent space of FRID, we perform UMAP dimensional reduction on the first $k$ rows of $V$. The dimensionality is set to 2 or 3 depending on the visualization target. To intuitively describe the contribution of each neuron on each latent axis, we assign coefficients to neuron based on the anchor it belongs to in the first K-means step.

**Modularized recurrent network simulation**

Firstly, we validated the effectiveness of FRID on the simulated dataset.

We leverage a recurrent neural network to generate simulation data as an important benchmark. Specifically, we consider the ensemble of neurons as a dynamical system, where the change rate of firing rate of each neuron is modeled as a stochastic differential equation, formulized as below:

$$\tau\frac{\text{d}r_{i}}{\text{d}t}=-r_{i}+\sum_{j}^{N} w_{ij}\tanh(r_{j})+n_{i}$$

The system is a self-organized system without any external inputs from outside, where $\tau$is the simulation time constant of neurons, $N$ is the total neuron number of the system, and $\tanh$is the hyperbolic tangent function as the nonlinearity. $r_{i}$ represents the firing rate of the single neuron$i$. $w_{ij}$ reflects the projection weight from neuron $i$ to neuron $j$, such that $[w_{ij}]$ , or $\mathbf{W}$ forms the connection matrix of the neuron population, and the second term on the right side of the stochastic equation represents how the other neurons in the system influence current neuron. $n_{i}$ represents the noise affecting neuron $i$ at certain time point, which is temporally dependent and is calculated using the following numerical sampling formula.

$n_{i}(t)=m_{i}(t)+[n_{i}(t-1)-m_{i}(t-1)]\cdot\exp(-\frac{\Delta t}{\tau_{\text{noise}}})$,

where $m_{i}(t)$ is sampled from a Gaussian distribution and is temporally independent, $\tau_{\text{noise}}$ is the time constant of the noise $n$.

The above continuous differential equation can also be rewritten as a discrete numerical form:

$\mathbf{r}(t+1)=\mathbf{r}(t)+\frac{\{-\mathbf{r}(t)+\mathbf{W}\tanh[\mathbf{r}(t)]+\mathbf{n}(t)\}\cdot\Delta t}{\tau}$,

where $\Delta t$ is the numerical sampling time step, $\mathbf{r}(t)$ and $\mathbf{n}(t)$ represent the vector form of the firing rates of different simulated neurons and noises applied to them at time point $t$.

The connection matrix is obtained by sampling from a Gaussian distribution. Before generating the simulated firing rate of the neuron population, we manually divide the population into certain groups, and then we generate the intra-group and inter-group connections by controlling the corresponding standard deviation of intra-connected and inter-connected weights. Intra-cluster weights have a higher probability of being larger, while inter-cluster weights tend to be smaller. Specifically, the intra weights follow the following formulas.

,

where $w_{\text{intra}}$ is certain single weight of intra-connected neurons, $C_{i}$ represents the number of the neurons in cluster $i$, $p$ represents the connection probability and $N_{c}$ is the number of clusters. $g$ controls the connection strength of the dynamical system and is usually set beyond 1.2 to keep the system chaotic. $x$ represents the random connection of neurons, sampling from a standard Gaussian distribution. And the inter weights follow as,

,

where $w_{\text{inter}}$ is a single weight of inter-connected neurons and generated independently. Obviously, the inter-connection is weaker than intra-connection.

After generating the connection matrix and noise, we used Euler method to simulate the neuronal population activity at each time stamp sequentially.

**Model comparison**

Except the proposed method, we consider a wide range of clustering methods as benchmark. We validate the effects of these methods on previously-mentioned simulation data.

To validate the methods, we first generated the simulated neuronal traces with $\text{d}t=0.1s,\tau=0.2s,\tau_{\text{noise}}=0.5s,T=6000,g=1.2,p=0,0.1,0.2,0.3\text{，}N_{c}=5$, and the neuron number of each cluster satisfied, where is the rounding operator.

As for the methods, for K-Means clustering, we set the centers to be 5, the criterion to be square Euclidean distance, and other parameters default in MATLAB. For Hierarchical clustering, we used the correlation as the criterion and the ward linkage, and we set centers to be 5. For the auto-encoder method, we construct an encoder and a symmetrical decoder, each of which contains sequentially a linear layer, a LeakyReLU layer as activation function, a dropout layer and ends with another linear layer. The hidden dimension of the first layer is set to be 1024 and the latent dimension 128. We use Adam as the optimizer and MSELoss as the loss function, and train the network for 300 epochs. Then we do inference to obtain the latent vectors of each neuron and apply a K-Means clustering method to group them into 5 groups, therefore labeling the neurons. For t-SNE combined with K-Means algorithm, we first reduce the dimension for the temporal dimension of calcium trace matrix into 3 using MATLAB built-in t-SNE function with default setting and then apply K-Means with a center of 5. For k-nearest neighbor graph, we modified previously described pipeline^1^ to eliminate supervised features. We first construct a binary adjacency matrix by finding each neuron’s k‐nearest neighbors using cosine similarity between neural traces, then we apply spectral clustering on it. For non-negative matrix factorization (NMF) methods^2^, we first normalize the values of the calcium trace matrix ranging from 0 to 1, then we do bootstrap. Specifically, we randomly sample temporal indices of the trace matrix with replacement to construct new bootstrapping matrices with the same shape as the raw matrix. After that, in a single round, we do NMF on the bootstrapping matrix, calculate the contribution of each neuron to construct binary matrix G and calculate the adjacency matrix as the product of G and the transpose of G. Final adjacency matrix is determined by averaging the adjacency matrices from each bootstrapping samples, and spectral clustering is applied on it to get the clustering results. For Leiden algorithm, we build a k-nearest-neighbor (k=20) graph using Pearson correlation coefficients as edge weights and symmetrize by taking the maximum. After that, we run the Leiden^3^ community-detection algorithm to adaptively search the resolution parameter (binary search from 1e−3 to 5.0) to obtain exactly 5 clusters. For FRID, we first use customized lite K-Means to select the anchors, with settings $M=1000,\alpha=0.1$, and the iteration is set to 100 and replications is set to 2. After that, we did the rest part of our proposed method and get the clustering results. When we got the clustering results, we did a best matching to the predicted clusters and ground truth using Hungarian algorithm. For FRID, we first use customized lite K-Means to select the anchors, with settings $M=1000,\alpha=0.1$, and the iteration is set to 100 and replications is set to 2. After that, we did the rest part of our proposed method and get the clustering results. When we got the clustering results, we did a best matching to the predicted clusters and ground truth using Hungarian algorithm.

For evaluation, we calculated the accuracy of the clustering result with ground truth and the normalized mutual information of the clusters of different methods. We also plotted the confusion matrices. Accuracy is calculated as $\text{Acc}=\frac{N_{R}}{N}$, where $N_{R}$ is the number of neurons which are correctly calculated. And the normalized mutual information (NMI) is calculated using the following formula.

Confusion matrix is created by counting how each predicted cluster identity is assigned and normalized with respect to the actual clusters.

In empirical data, we also calculated the spatial separation degree (SSD) of the clusters. The index is defined by calculating the averaged distance within cluster divided by the averaged distance between cluster.

$SSD=\frac{1}{N}\sum_{i=1}^{N} \frac{(N-C_{K_{i}})\sum_{K_{i}=K_{j}} d(i,j)}{C_{K_{i}}\sum_{K_{i}\neq K_{j}} d(i,j)}$,

where $K_{i}$ is the cluster id of neuron $i$, $C_{K_{i}}$ represents the number of the neurons in cluster $K_{i}$, $d(i,j)$ is the Euclidean distance between neuron $i$ and neuron $j$.

**Light field imaging and calcium extraction**

For real data acquisition, we use RUSH3D^4^, a scanning light-field microscopy to record 3D neural imaging data. The system has a uniform single-neuron resolution of $2.6\times2.6\times6\text{ μm}^{3}$ across a volume of $8\times6\times0.4\text{ mm}^{3}$. The imaging frequency is set to 10Hz.

For calcium extraction, we used sparse-seeded iteration demixing (SSID), a neuron extraction pipeline to obtain calcium traces^4,5^. Specifically, the light-field data is first resized and rotated to ensure each microlens encompasses 15x15 sensor pixel, and then realigned, transforming the unprocessed measurements of the RUSH3D into phase space. A captured frame can be characterized as $F(x, y, u, v, t)$, where $(u, v)$ indicate the relative position of sensor pixels after each microlens, corresponding to the angular coordinates, and $(x, y)$ signify the center position of each microlens, corresponding to the spatial coordinates. In the context of scanning LFM, samplings from an identical view $(u, v)$ within a scanning duration are collated to yield high-resolution spatio-angular measurements $F_{h}(x, y, u, v, t)$, which is achieved by combining all the frames from the same view in the order of the scanning positions throughout an entire scanning cycle (a 3 × 3 scanning pattern is used in our recording). A sliding window technique is incorporated in the temporal domain, ensuring the preservation of the majority of temporal information.

Next, a multi-scale background removal algorithm is applied to increase the signal-background ratio. the view measurements are firstly down sampled for initiating a quick yet axially extended 3D reconstruction to adequately model only the background without foreground signals. The background planes are reconstructed residing on either side. Subsequently, these background planes are back-propagated into the light field space, resulting in background-only views. Then, those background views were differentiated with the original field captures to generate background-removed light field frame. After that, we do motion registration for the realigned data via the open-source NormCorre^6^ algorithm and calculate the standard deviation (STD) of the video over time. Then we reconstruct one summary STD 3-dimensional image with calibrated system point spread function with wDAO, followed by neuronal segmentation. After neuronal footprints are obtained, we project them forward onto different views to initialize the 2D neuronal footprint and temporal trace, and then iteratively demix to enhance the traces, involving only 13/255 of the comprehensively captured angular data. The spatial and temporal components were alternately updated whilst maintaining the other constant, following the non-negative matrix factorization (NMF)

**Neural signal analysis**

**Preprocess**

Calcium traces obtained by SSID are a result of NMF, representing the normalized fluorescence intensity of the neuron. Before using it for FRID analysis and decoding, several preprocessing steps are conducted. Firstly, in cross-session analysis, we remove low-frequency drift of fluorescence intensity by subtracting the mean-filtered trace of each neuron. The size of the filter is set to 20 s. Then, each neuron trace is z-scored to achieve a mean of 0 and std of 1.

**Neuropixels data preprocessing and FRID analysis**

One publicly available Allen Brain Observatory Visual Coding Neuropixels recording session^7^ was used for the electrophysiological proof-of-concept analysis. Units were filtered according to the presence ratio, amplitude cutoff, inter-spike-interval violation rate and mean firing rate. Spike trains recorded during repeated presentations of nature movies were divided into 100-ms bins and converted into firing-rate traces. Each movie presentation was smoothed independently along the temporal dimension using a one-dimensional Gaussian kernel with a standard deviation of 100 ms. The processed movie presentations were subsequently concatenated along the temporal dimension without smoothing across presentation boundaries, and each neuronal trace was z-scored over the concatenated sequence.

FRID was applied to the resulting neuron-by-time activity matrix. The number of clusters was fixed at eight for this proof-of-concept visualization. We used 200 anchors, a regularization parameter of 0.1, trace normalization, and two repetitions for anchor initialization. In the activity heatmap, neurons were ordered according to their FRID cluster identities, with black horizontal lines indicating cluster boundaries. Gray vertical dashed lines indicate the boundaries between concatenated natural-movie presentations.

Pupil area was interpolated to the same 100-ms temporal bins, smoothed using a Gaussian kernel with a standard deviation of 200 ms and z-scored before visualization. Pupil information and anatomical annotations were not provided to FRID. Gray vertical dashed lines in Extended Data Fig. 16f and g indicate the boundaries between concatenated natural-movie presentations.

For anatomical characterization, the fraction of neurons assigned to each annotated brain region was calculated separately within each cluster. To visualize neuronal relationships in the FRID representation, the similarity matrix was calculated using the cosine similarity between the absolute anchor-coefficient profiles of each pair of neurons.

**Representative response and supervised neuron selection**

The trial-averaged response of each neuron is calculated by aligning the start time of each stimulation trial. The averaged activity of 1 s pre-stimulus is considered as baseline and subtracted from single-trial responses.

To interpret the functional role of each cluster, we calculated the distribution of selective neurons in functional clusters (Fig.3f). Neurons are defined as selective to the specific image (chord) if it passes a significance test. A two-sided Wilcoxon rank sum test is performed between the responses of a specific image (or chord) and all other images (or chords). Meanwhile, the average response of the specific image (or chord) is required by the maximum among all other possible images (or chords). The *p* threshold is set as 5×10^-4^ in visual task and 5×10^-3^ in auditory task.

**Unique PCA analysis**

We first calculate the basis of each subspace $\bar{U}_{k}$ produced by FRID with singular value decomposition (SVD).

$$X_{k}=U_{k}\sum_{k}V_{k}^{T}$$

where contains $N_{k}$ neurons that belong to Cluster #k, each with T sampling time points. $U_{k}$ and $V_{k}$ are unitary matrices. To reduce the noise and computational load, we keep the first $r_{k}$ left singular vectors, where the singular values accounts for 80% of total variance.. Then we extract the common components appears in at least half of the subspace by performing SVD on the concatenated basis

We select the basis $b_{i}$ in $B$ whose singular value $\sigma_{i}>\sqrt{K/2}$, which means they are shared by at least half of the clusters, denoted as shared PC,. This part may be related to the low-frequency spontaneous activity of the mice and the high frequency mechanical noise (Extended Data Fig.14f). Then we remove the intersect of $\bar{U}_{k}$ and $B_{^{s}}$ by projecting $\bar{U}_{k}$ to $B_{^{s}}$.

$$\tilde{U}_{k}=\bar{U}_{k}-B_{^{s}}B_{^{s}}^{T}\bar{U}_{k}$$

Now each column in $\tilde{U}_{k}$ contain a unique PC of this specific cluster, preserving the representative traces of each cluster, uniquely preserved by this cluster.

**Decode of task variables**

To evaluate the coding ability of the functional clusters, a KNN classifier is used as the decoder in the multi-sensory presentation task. The population vector of each stimulation is calculated by subtracting the mean response of 1 s pre-stimulus response from the mean response of 1.5s post-stimulus. We used the Pearson correlation between the population vectors of each trial as the distance metric in KNN. In visual experiments, to compare the decoding accuracy between different clusters and visual cortex, we randomly sample 200 neurons from each cluster and repeated the sampling for 10 times. We set K=5 and used 100-fold validation. In multi-sensory protocol, to compare different clusters whiling preserving the number of neurons in the cluster, we randomly sample 90% of neurons from each cluster and repeat the sampling for 10 times. we set K=5 and used 100-fold validation. In the Go/No-Go protocol, we set K=3 and used 30-fold validation.

Some continuous behavioral variables are extracted from the face camera and Bpod system. The lick, reward, and air puff activity traces are obtained by applying a Gaussian filter with $\sigma=1.5\text{ s}$ to the binary event train. The PC of motion is calculated by performing PCA to the vectorized and down-sampled video recording of the face camera. The influence of spontaneous movement on the populational activity of neurons is evaluated via a linear model that fits the PCs of functional cluster with PCs of motion (Extended Data Fig.22d). To decode the continuous variables (Fig.6f), we performed Lasso regression using *lasso* function in MATLAB to avoid overfitting. Number of Lambda values is set to 20, and 5-fold validation is used to evaluate the decoding performance. The minimal mean square error (MSE) between the fitted behavior variable and the ground truth of all Lambda values is used as the evaluation metric. The *p*-value of the functional cluster decoding fidelity is obtained via a shuffled test. We randomly sample $C_{i}$ neurons from all neurons and decode the behavior variable out of these neurons. $C_{i}$ is the number of neurons in cluster $i$. The shuffled sampling and decoding are repeated for 100 times, and the percentile of the empirical MSE among shuffled MSE is considered as *p* value. To indicate the tail of the distribution, we denote *p_1_* as the ascended percentile and *p_2_* as the descended percentile, where *p_1_* = 1- *p_2_*. In Fig.6f, the block is colored based on the smaller value of *p_1_* and *p_2_*. The red blocks (*p_2_* < 0.05) indicated significantly better decoding from empirical cluster, and blue ones (*p_1_* < 0.05) indicates significantly worse decoding ability.

**Calcium imaging**

We used mice expressing GCaMP6s in layer 2/3 cortex (Ai162^8^ x Rasgrf2-2A-dCre) to capture cortex-wide calcium activities at single-neuron resolution. Mice were held in plastic cages with standard temperature and humidity. Trimethoprim (TMP, 0.25 mg/g, Sigma) is injected for 2 days to induce the expression of GCaMP6s.

The craniotomy is performed on adult mice aged 8-10 weeks. Briefly, mice were anesthetized with 1-2% isoflurane. The scalp was removed to expose the skull. An 8 mm craniotomy was made by skull drill on the right hemisphere. A custom-designed titanium headpost with a glass coverslip was then implanted to allow optical access to the cortex surface. The mice recovered for at least 2 weeks before the fluorescence imaging.

During calcium imaging, mice were head-fixed on a home-made holder. The headbar was mounted on a 5-axis motorized stage for accurate x-y-z translation and tilt adjustments. The cranial window was adjusted to orthogonally to the optical axis of the mesoscope. The focal plane was set to be targeted at the layer 2/3 of labelled neurons. Calcium imaging was performed at a frame rate of 10 Hz.

**Retinotopic mapping**

To register multi-brain areas to CCF atlas, a retinotopic mapping pipeline was employed to map the visual cortex regions. Other brain areas are registered relative to the visual cortex. We used a custom-built polarization-gated wide-field imaging system, which is similar to those previously described elsewhere^9^. 528 nm green light is used as the detection wavelength. We followed a standard retinotopic mapping procedure^10^. Briefly, mice are slightly anesthetized with ~0.6% isoflurane. A spherically-corrected drifting checkerboard was presented on a 27-inch screen positioned 15 cm away from the left eye of the mice. The periodical stimulation was repeated for 40 trials. The gradient map of the receptive field was computed using the first harmonic component of the Fourier series of the trial-averaged response of each pixel. A semi-manual program was used to register the retinotopic map to an oblique projected CCF atlas. The projection angle ranges from 25°to 45°, depending on the retinotopic map of each subject. Fluorescence imaging results were aligned to intrinsic images using vescular morphology as structural guidance.

**Visual, auditory and multi-sensory passive presentation**

For the evaluation of our clustering pipeline, several passive sensory exposure protocols were employed, including visual stimulation, auditory stimulation and multi-sensory stimulation. Mice were placed in a soundproof room to ensure stability during the experiment. Their heads were fixed using a customized head holder, which allowed precise alignment under the scanning light-field microscope for monitoring neural activity. The delivery of sensory stimuli were controlled and synchronized using Bpod (Sanworks) system.

**Visual stimulation**

Visual stimuli were presented on an 8-inch LED screen with a resolution of 1920x1200 positioned 15 cm from the mouse's left eye. The R and G channels of the LED screen were turned off to avoid crosstalk between the fluorescence and the screen light. During visual experiments, 12 kinds of grating patterns and 12 natural images were displayed in pseudorandom order, each lasting for 1.5s. The drifting grating patterns consisted of 12 orientations spaced 30°apart, ranging from 0°to 330°, and the inter-stimulus interval (ISI) between two trials varied from 5s to 7s. Each image is presented for 10 repeats.

**Auditory stimulation**

4 different chords were used as auditory stimuli and delivered through a speaker positioned 40 cm from the mouse's ear. The composition of the chords is as previously described^11^. The sound level was approximately 60 dB. Each chord lasts for 2 s and the ISI ranged from 6 s to 8 s. The four chords were presented in a pseudorandom order, and each was repeated 20 times.

**Multi-sensory stimulation**

During the multi-sensory experiment, three kinds of stimuli were used: visual, auditory, and tactile. The visual stimulus consisted of static grating patterns with 4 orientations, 0°, 45°, 90°, 135°, which lasts for 1s. And the ISI is 4 s to 8 s. The auditory stimulus was pure tones with 1, 4, 8, 16 kHz frequency. The tactile stimulus was delivered using a controlled air-puff system that emitted a brief burst of air directed at the face of the mouse. Each air puff lasted for 1s, with an ISI of 4 s to 8 s. The timing and delivery of air puffs were synchronized with the visual and auditory stimuli to assess multimodal integration.

These three kinds of stimuli were presented either simultaneously or separately within a single trial. In each trial, each type of visual and auditory stimulus appeared with a probability of 20% each, while the remaining trials contained no visual or auditory stimulus. The air puff was randomly presented in 50% of trials. The total trial number is 280. The duration of a trial is 1 second and the inter-trial interval last for 4 to 8 seconds.

**Pavlov training**

We trained the head-fixed mice to Pavlov and Go/No-Go task in a progressive way. Adult mice, aged around 12 weeks were used in the experiment. All mice were housed in standard laboratory conditions with a 12:12-hour light-dark cycle and ad libitum access to food.

The training stages followed protocols similar to those described elsewhere^12,13^. Briefly, mice are water restricted to ~80% of the initial body weight. In the first stage (habituation), mice were placed in a head-fixed position using a custom-designed apparatus that allowed for stable recording of their behavior while maintaining comfort. The mice were trained to free lick water from the lick port for 2-3 days. In the second stage (Pavlov), the mice were exposed to the Go cue. After a short delay (0.2 s), a drop of water (~5μL) was delivered through the lick port. The mice learn the pairing between conditional stimulation and unconditional stimulation through repeated cue-reward pairing. Mice that reliably lick at the cue presentation continue to the Go/No-Go training.

**Go/No-Go training**

The Go/No-Go training session consisted of a series of trials, each comprising the following components. Each trial began with the presentation of an auditory cue lasting for 2 s. The Go chord indicated the opportunity to earn a reward, whereas the No-Go chord signaled potential punishment if a false alarm lick is made. Following a 0.2 s delay after cue offset, the response window opened. In the Go trials, the mice were required to lick the port within 1 s to receive a reward of 5μL clean water. A successful lick during the Go trials resulted in an immediate reward delivery. In No-Go trials, mice were required to withhold licking during the 1 s to avoid an air puff to the face and a white noise delivery. The inter-trial interval was 6 s to 8 s. An additional time-out of 3 s was used to suppress false alarm licks in No-Go trials.

Mice underwent 5 to 10 days of training sessions, during which they performed the Go/No-Go tasks. Each session consisted of 240 trials with Go and No-Go trials presented in pseudorandom order to prevent predictability.

**Induction of photothrombotic stroke model**

Mice were anesthetized by intraperitoneal injection of avertin (307 mg/kg, dissolved in saline at 19.2 mg/ml; Sigma-Aldrich, CAS 75809) and anesthesia depth was verified by absence of toe pinch reflex before proceeding. The cranial window was gently disinfected using an alcohol swab and allowed to dry completely. The mouse was then immobilized in a stereotaxic frame, and the target region for photothrombosis was identified under microscopic guidance. A circular region of interest (300 pixels in diameter, approximately 1 mm) was delineated using Fiji software.

Rose Bengal (50 mg/kg, dissolved in saline at 10 mg/ml; Sigma-Aldrich, #330000) was then administered via tail vein injection and allowed to circulate systemically for 5 minutes. The anesthetized mouse was positioned under the laser system, and the designated region was irradiated for 5 minutes using a 561 nm laser at an intensity of 100 mW/mm². Successful model induction was confirmed by microscopic verification of complete blood flow cessation in the irradiated area vessels following photothrombosis.

1	Hirokawa, J., Vaughan, A., Masset, P., Ott, T. & Kepecs, A. Frontal cortex neuron types categorically encode single decision variables. *Nature* **576**, 446–451 (2019). <https://doi.org/10.1038/s41586-019-1816-9>

2	Nagayama, M. *et al.* Detecting cell assemblies by NMF-based clustering from calcium imaging data. *Neural Netw* **149**, 29–39 (2022). <https://doi.org/10.1016/j.neunet.2022.01.023>

3	Traag, V. A., Waltman, L. & van Eck, N. J. From Louvain to Leiden: guaranteeing well-connected communities. *Sci Rep* **9**, 5233 (2019). <https://doi.org/10.1038/s41598-019-41695-z>

4	Zhang, Y. *et al.* Long-term mesoscale imaging of 3D intercellular dynamics across a mammalian organ. *Cell* **187**, 6104–6122 e6125 (2024). <https://doi.org/10.1016/j.cell.2024.08.026>

5	Cai, Y., Wu, J. & Dai, Q. Review on data analysis methods for mesoscale neural imaging in vivo. *Neurophotonics* **9**, 041407 (2022). <https://doi.org/10.1117/1.NPh.9.4.041407>

6	Pnevmatikakis, E. A. & Giovannucci, A. NoRMCorre: An online algorithm for piecewise rigid motion correction of calcium imaging data. *J Neurosci Methods* **291**, 83–94 (2017). <https://doi.org/10.1016/j.jneumeth.2017.07.031>

7	Siegle, J. H. *et al.* Survey of spiking in the mouse visual system reveals functional hierarchy. *Nature* **592**, 86–92 (2021). <https://doi.org/10.1038/s41586-020-03171-x>

8	Daigle, T. L. *et al.* A Suite of Transgenic Driver and Reporter Mouse Lines with Enhanced Brain-Cell-Type Targeting and Functionality. *Cell* **174**, 465–480 e422 (2018). <https://doi.org/10.1016/j.cell.2018.06.035>

9	Song, X. *et al.* Mesoscopic landscape of cortical functions revealed by through-skull wide-field optical imaging in marmoset monkeys. *Nat Commun* **13**, 2238 (2022). <https://doi.org/10.1038/s41467-022-29864-7>

10	Zhuang, J. *et al.* An extended retinotopic map of mouse cortex. *Elife* **6**, e18372 (2017). <https://doi.org/10.7554/eLife.18372>

11	Wang, M. *et al.* Single-neuron representation of learned complex sounds in the auditory cortex. *Nat Commun* **11**, 4361 (2020). <https://doi.org/10.1038/s41467-020-18142-z>

12	Guo, Z. V. *et al.* Procedures for behavioral experiments in head-fixed mice. *PLoS One* **9**, e88678 (2014). <https://doi.org/10.1371/journal.pone.0088678>

13	Ebrahimi, S. *et al.* Emergent reliability in sensory cortical coding and inter-area communication. *Nature* **605**, 713–721 (2022). <https://doi.org/10.1038/s41586-022-04724-y>

# Supplementary Note

**Toy models to explain the principle and insight of FRID**

Here, we give a toy simulated example to explain the insight of sub-space clustering and its difference with correlated-firing-based methods. The simulated system containing 8 neurons that belong to 2 modules. Neurons are densely connected by complex connectivity patterns in each module. And neuron #4 and neuron #5 form a narrow bridge between these two modules (Figure N1a). Using neural membrane dynamic function1 (Method), we generated the spontaneous activity of neurons (Figure N1b). In this simple example, we cover 2 typical cases. The first case is that because of inter-module connectivity, neurons form close firing pairs (Neuron #4 and Neuron #5). But actually, they do not belong to the same module. The second case is that neurons from the same close community might be highly dissimilar (Neuron #8 and Neuron #6). This is because the complex connectivity produces chaotic dynamics inside the module, which cannot be revealed by pairwise correlation or causality and leads to highly heterogeneous firing patterns. These two circumstances could all cause the failure of correlated-firing-based methods. On the contrary, FRID does not require neurons to behave similarly to be included in the same cluster. Instead, FRID approximates the relationship between neurons using quadratic optimization and then discovers the consistent subspace the neurons belong. These subspace structures could expand a large range of neural feature space (Figure N1c), overcoming the chaotic feature that the system exhibits. This is the core difference between FRID and other methods.

**Figure N1 | A simple toy model for conceptual clarification of the fundamental difference between FRID and other synchronized-firing-based models. (a),** Diagram of the example network containing 2 modules connected by 2 neurons. **(b)**, The simulated spontaneous activity of the neurons in the network. **(c)**, The activity of the neurons in **(b)** is projected into an MDS space. The distance matrix used for dimension reduction is the pairwise Pearson correlation. The yellow and green shading is the ground truth of the module division, which FRID could recognize because of the low subspace (a line-like 1D space on the 2D MDS space) structure. And the dashed circles show two typical cases that could cause the failure of synchronized-firing-based methods.

Another toy example is given to show why co-tuning could be misleading in clustering. Assume Neuron #1 to Neuron #4 belong to the visual cortex (VIS), and Neuron #5 to Neuron #8 belong to the somatosensory cortex (SSp). Within each functional cluster, neurons are densely connected, and between these two clusters, only Neuron #4 and Neuron #5 are connected, forming a narrow information pathway (Figure N2a). Now we inject input stimulus into the network through the corresponding neural population. Visual pulses are injected into the activity of N#1 – N#4 with the same projection weight, and whisker stimulus input is injected into the activity of N#5 – N#8 with the same projection weight. The firing rate of this network is generated with the neural membrane dynamic function1. More than 100 pulses are presented, and the trial-averaged response of each neuron could be calculated (Figure N2b). When examining the trial-averaged activity of the 4 neurons in VIS, although they are all related to the visual stimulation with the same strength, we found some neurons show ‘selectivity’ very strongly (N#4, N#2, N#1), but some do not (N#3). And one neuron from the SSp (N#6) shows considerable response after a certain temporal delay. Similarly, in whisker stimulus response, one neuron (N#4) from VIS shows considerable activation after the whisker stimulus although actually it is in the VIS module. The reason for this counter-intuitive result is that neural activities are not only driven by their encoding sensation, but also largely influenced by their connected neighbors. To give an example, N#7 receives strong inhibition from its neighbor N#8 and N#5, which offsets the activation from external whisker stimulation, leading to its flattened tuning curve. But this should not exclude N#7 from the SSp module, because it is both strongly connected with other SSp neurons and also receives whisker-related input. Imagine next time, when the input stimulus is no longer uniform, but specifically targeted at N#7 (for instance, from multi-whisker stimulus to single-whisker stimulus), the inhibition property of other neurons in SSp vanishes, and the tuning of N#7 will thus be strong. And for experiments, it is just impossible to cover every possible combination pattern of stimulus with infinite stimulus trials. So, the trial-averaged response of one neuron only tells the overall effect of a certain external stimulus, but it contains both direct input and indirect influence of other nodes. The latter is often more intrinsic, complicated, and non-linear. This is exactly what FRID focuses on, the mutual relationship between one neuron and the population. The tuning property of the neuron may change based on the current stimulus pattern, but the relationship between neurons is relatively stable. For instance, N#7 always receives inhibitive influence from N#5 and N#8, and always receives positive influence from N#6, which is determined by the effective synaptic connectivity. FRID first estimates this stable relationship, and naturally leads to the resulting tuning property afterwards. Calculating the trial-averaged response can tell us about the approximate property of the cluster, but definitely not accurate enough to describe the role of the neuron in the overall network. So, if using some supervised method, neurons like N#7 and N#3 could be incorrectly grouped.

**Figure N2 | An toy example with external stimulus input. (a),** Diagram of the example network containing 2 modules connected by 2 neurons. **(b)**, The simulated spontaneous activity of the neurons in the network.

# Supplementary Table 1

| **Category** | **Method name** | **Key insight** | **Running time (6000*6000)** | **Accuracy**  **(P = 0.1)** |
| --- | --- | --- | --- | --- |
| Subspace based | FRID | Group neurons carrying high mutual information (lands in the same subspace) into the same functional cluster | 900 s | 0.9997 ±0.0005 |
| Co-firing based | K-means | Partition neurons with similar co-firing patterns | 75 s | 0.2259  ±0.0068 |
|  | Hierarchical clustering | Build a tree of nested clusters with similar co-firing patterns. | 20 s | 0.2496  ±0.0097 |
| Similar low-dimensional feature-based | UMAP | Group neurons with similar UMAP manifold embeddings | 2.5 s | 0.2427  ±0.0254 |
|  | t-SNE | Group neurons with similar t-SNE manifold embeddings | 200 s | 0.2336  ±0.0102 |
|  | ICA | Group neurons with the features in the mixing matrix | 25 s | 0.2278  ±0.0096 |
|  | Auto encoder | Group neurons with features extracted by a deep autoencoder. | 230 s | 0.2282  ±0.0074 |
| Pairwise- similarity-based | NNG  (Hirokawa, 2019) | Construct a nearest-neighbor graph and detect strongly connected subcomponents as clusters | 100 s | 0.2501  ±0.0103 |
|  | NMF (Nagayama, 2022) | Use non-negative matrix factorization to create a similarity matrix and group neurons with spectral clustering | 4,000 s | 0.2280  ±0.0065 |
|  | Granger causality * | Use pairwise neuronal Granger causality as a similarity matrix and group neurons with K-Means clustering | >100,000 s *  (> 28 h) | 0.2935  ±0.0180 * |
|  | Leiden (Traag, 2019) | Construct a kNN/SNN graph and maximize a community quality function with the Leiden refinement–aggregation algorithm to obtain well-connected clusters. | 60 s | 0.2393  ±0.0104 |
| Spiking- model-based | Bayesian clustering  (Wei, 2022) * | Cluster neurons that share consistent low-dimensional latent factors by a Bayesian mixture of dynamic Poisson factor analyzers/mixture of Poisson linear dynamical system | >110,000 s *  (> 28 h) | 0.3102 ±0.0258 * |
|  | mixPLDS  (Buesing, 2014) * |  |  |  |
|  | FEU  (Lin, 2024) | Utilize a Dirichlet process mixture of state-space models to partition neurons into different ensembles characterized by interpretable parameters. | >500,000 s *  (> 139 h) | 0.2975 ±0.0218 * |
| Co-tuning based | TCA  (McGuire, 2022) | Use tensor component analysis to assign neurons to the cluster with the highest tuning pattern. Supervised by the trial division. | - | - |

**Extended Data Table 1 | Comparison of key insight, computational load, and accuracy on simulation datasets for different methods.** * stands for the methods whose computational complexity is extremely high and whose performances are tested on down-sampled datasets. The computational loads of these methods are estimated for the standard datasets based on the running time of the down-sampled datasets and their own computational complexities. The down-sampled dataset has a shape of around (120, 600).

# Extended Data Figure 1

**Extended Data Fig. 1 | Hyperparameter evaluation and cross-session subspace clustering pipeline. (a-b),** Accuracy and normalized mutual information via different magnitudes of alpha and anchor number. **(c),** An example affinity matrix in simulated dynamical system. **(d)**, Cross-session subspace clustering pipeline. Sessions are concatenated to select anchors from multi-session trace. Anchors are then used to calculate the affinity in each session. Subspaces are aligned by concatenate the affinity matrix generated from multi-session anchor. Multi-session similarity matrix is then obtained, upon with spectrum clustering is performed.

# Extended Data Figure 2

**Extended Data Fig. 2 | Evaluation of competing methods in identifying functional clusters through numerical simulation of modularized dynamical systems.** **(a)** Comparison of the confusion matrices obtained by FRID, hierarchical clustering, K-Means clustering, auto-encoder (AE) with K-Means, t-SNE with K-Means, spectral clustering, NNG (Hirokawa, 2019), NMF (Nagayama, 2022), ICA with K-Means clustering, UMAP with K-Means under different inter-cluster connection strength P increasing from 0 to 0.5. **(b)** Comparison of the accuracy and normalized mutual information (NMI) between inferred functional cluster results and the actual modules defined by simulation datasets under different inter-cluster connection strength P, ranging from 0 to 0.3. **(c)** Same as **a**, but on a demonstrated down-sampled dataset due to computational complexity. Results of Bayesian clustering (Wei, 2022), FEU (Lin, 2024), and Granger Causality followed by K-Means are shown. **(d)** Same as **b**, but on a down-sampled dataset. **(e, f)** Performance of K-means clustering with different parameters under P = 0.0125. n = 10 repeats, mean ± s.e.m..

# Extended Data Figure 3

**Extended Data Fig. 3 | Choice of the number of clusters and individual consistency. (a)** The largest 20 singular value of the affinity matrix, different panels show different ground-truth cluster number. Each diamond shows a repeated run of FRID, and the blue shading shows the std between runs (*n*=5). **(b)** Example confusion matrixes when the assumed number of clusters (3-7) is not correct (5). Bottom, the clusters are merged according to their best-matched cluster; by merging the clusters, the accuracy of clustering is recovered. **(c)** The accuracy and normalized mutual information (NMI) of raw and merged clustering result. Miscalculated cluster number may split one cluster to several clusters or combine several clusters into one, but hardly mix unrelated clusters. Each diamond shows a repeated run of FRID, and the shading shows the std between runs (*n*=5). **(d)** Singular value spectrum in 4 mice. **(e)** FRID clustering results with 4 different mice. **(f)** Density of neurons in 4 different mice. **(g)** The number of neurons in the visual cortex in 4 different mice.

# Extended Data Figure 4

**Extended Data Fig. 4 | Large scale recording with real-time, ultra-large-scale, high-resolution 3D mesoscope (RUSH3D). (a)** Maximum intensity projection of the reconstructed 3D std volume in XY, XZ and YZ plane. **(b)** The gradient map of retinotopic receptive field. **(c)** The alignment of retinotopic map with CCF atlas. **(d)** The registered cranial window over CCF atlas. **(e)** the de-backgrounded vessel image overlayed with CCF atlas. **(f)** Extracted neuron footprints overlayed with CCF atlas. **(g)** Cortex area distribution of neurons. (*n*=5). Inset: the reference brain areas and the name of the brain areas.

# Extended Data Figure 5

**Extended Data Fig. 5 | Evaluation of different methods on experimental datasets collected under 3 different protocols. (a)** Comparison of NMI between spatially defined CCF brain areas and identified functional clusters obtained by FRID, K-means clustering, hierarchical clustering, NNG (Hirokawa, 2019), UMAP with K-Means, and ICA with K-Means. The white dot represents the median, and each filled dot represents an individual run. We used 3 protocols and 3 runs for each protocol with a specific cluster number. **(b)** Same as a, but showing spatial separation degrees between spatially defined CCF brain areas and identified functional clusters. **(c)** Spatial distributions of functional clusters obtained by FRID, K-means clustering, and hierarchical clustering, NNG (Hirokawa, 2019), UMAP with K-Means and ICA with K-Means. **(d)** Same as **a**, b, but on a down-sampled demonstration dataset due to computational complexity. Results of FRID, Bayesian clustering (Wei, 2022), FEU (Lin, 2024) and Granger Causality with K-Means are shown. **(e)** Spatial distributions of functional clusters obtained by Bayesian clustering (Wei, 2022) and FEU (Lin, 2024) and Granger Causality with K-Means.

# Extended Data Figure 6

**Extended Data Fig. 6 | The cluster number in empirical data. (a)** The largest 20 singular value in 3 different behavior protocols. **(b)** Distribution of functional clusters overlayed with CCF atlas with increased number of clusters. Each row shows one behavioral protocol. **(c)** The change of the cluster assignment of each neuron with the increase of assumed cluster number.

# Extended Data Figure 7

**Extended Data Fig. 7** | **Spatial temporal functional map in visual presentation task. (a)** the spatial footprint of neurons in every functional cluster. **(b)** Calcium responses of neurons in each functional type. Arrows at the bottom indicate the start of each trial, the color of the arrow stands for the image type. **(c)** Trial-averaged response of neurons in each functional cluster, trials presenting grating and trials presenting natural images are shown respectively.

# Extended Data Figure 8

**Extended Data Fig. 8 | uPC visualization of each cluster under visual presentation and the clustering result under different circumstances.** **(a)** In each sub panel, the left part shows the uPCs, the right part shows the corresponding trial-averaged traces, where solid lines represent nature images and dashed lines represent the gratings. The gray vertical dashed line represents the start of the stimulus. Different colors represent different nature images or gratings. **(b)** clustering result of k-means using the peri-stimulus response and the whole-session neural activity. **(c)** The clustering result in the same mouse under resting state and visual presentation protocol.

# Extended Data Figure 9

**Extended Data Fig. 9 | Spatial temporal functional map in chord presentation task. (a)** the spatial footprint of neurons in every functional cluster. **(b)** Calcium responses of neurons in each functional type. Arrows at the bottom indicate the start of each trial, the color of the arrow stands for the chord type. **(c)** Trial-averaged response of neurons in each functional cluster, different types of trials are shown respectively.

# Extended Data Figure 10

**Extended Data Fig. 10 | uPC visualization of each cluster under chord presentation.** In each sub panel, the left part shows the uPCs, the right part shows the corresponding trial-averaged traces. The gray vertical dashed line represents the start of the stimulus. Different colors represent different chords.

# Extended Data Figure 11

**Extended Data Fig. 11 | Spatial temporal functional map in Pavlov protocol. (a)** the spatial footprint of neurons in every functional cluster. **(b)** Calcium responses of neurons in each functional type. Arrows at the bottom indicate the start of each trial, the color of the arrow stands for the chord type. **(c)** Trial-averaged response of neurons in each functional cluster.

# Extended Data Figure 12

**Extended Data Fig. 12 | uPC visualization of each cluster under Pavlov protocol.** In each sub panel, the left part shows the uPCs, the right part shows the corresponding trial-averaged traces. The two gray vertical dashed lines represent the start of the stimulus and the start of the decision window respectively. Both hit and miss trials are included.

# Extended Data Figure 13

**Extended Data Fig. 13 | Analysis on the clustering result of visual presentation protocol. (a)** The distribution of neurons on CCF atlas in cluster 6 and 7. **(b)** The number of selective neurons to 12 grating images (G1-G12) and 12 natural images (NI1- NI12). **(c)** Response curves to 24 different visual stimulations of 24 example neurons from cluster 6 and 7. The dashed lines indicate the starting point of the visual stimulus. **(d)** The correlation of the first 5 PCs of each of the functional clusters. **(e)** The shared PCs between clusters and the PCs extracted from the facial recording video captured with Calcium imaging. **(f)** The decoding accuracy of increasing number of neurons randomly sampled from VIS area and FRID-defined visual-related cluster. The shading stands for s.e.m., 4 mice, each with 5 technical repeats of FRID and 10 bootstrapped sampling of neurons.

# Extended Data Figure 14

**Extended Data Fig. 14 | Example neurons from the visual presentations task and the result of auditory presentation task. (a)** The first 10 uPCs of cluster #7 in the visual experiment. Single-trial activity and trial-averaged activity are shown on the left and the right. Each shading in the left shows the visual stimuli colored by its identity. The same color is used in the trial-averaged response on the right. Grating (G) is plotted with dashed lines, and natural image (NI) is plotted with solid lines. The vertical gray dashed line shows the start of the visual stimulation. **(b), (c)** Same as **a**, but showing the VIS cortex and cluster 6. **(d)** The location of 10 example neurons in Figure 3f, and the overall location of cluster #7 neurons (inset). **(e)** The proportion of specific-chord-selective neurons in each functional cluster in chord-presentation task. **(f)** The number of selective neurons to chord 1-4. **(g)** The proportion of chord-selective neurons in each of the 7 clusters.

# Extended Data Figure 15

**Extended Data Fig. 15** | **Screenshot of the graphical user interface (GUI) of FRID.** The right side of the GUI lays the requirements and inputs, including the data path, save path, and neuronal position path. Parameters of FRID, such as cluster number, sparsity penalty (alpha), and anchor number could be input. The left side of the GUI shows the visualization of selected output uPC (top) and the spatial footprints of the specific cluster (bottom).

# Extended Data Figure 16

**Extended Data Fig. 16 | Evaluation of the framework on other datasets. (a)** Functional map of a 3D drosophila brain. Voxel level Calcium signal is recorded by a two-photon microscopy, each voxel is treated as a node. **(b)** Functional map from whole-brain recording of zebra fish. **(c)** Confusion matrix between the functional cluster and the session ID in a pseudo mice dataset. **(d)** FRID-sorted neuronal activity from one publicly available Allen Brain Observatory Neuropixels recording session. Spike trains recorded during repeated presentations of natural_movie_one were converted into 100-ms binned firing-rate traces, Gaussian-smoothed and z-scored before FRID analysis. Neurons are ordered according to their FRID cluster identities, with black horizontal lines indicating the boundaries between the eight identified clusters. Gray vertical dashed lines indicate the boundaries between concatenated natural-movie presentations. **(e)** Simultaneously recorded pupil area aligned to the neuronal activity in a and expressed as a z-score. **(f)** Anatomical composition of the eight FRID clusters. Each row shows the fraction of neurons assigned to the indicated brain regions after excluding unspecified and aggregated anatomical categories and renormalizing the remaining regional fractions within each cluster. **(g)** Similarity matrix derived from the absolute FRID anchor-coefficient profiles, with neurons ordered according to their cluster identities. Black horizontal and vertical lines indicate cluster boundaries.

# Extended Data Figure 17

**Extended Data Fig. 17 | Spatial distribution of functional clusters in multi-sensory task. (a)** The cortex area distribution of example functional clusters. **(b)** The distribution of neurons on CCF atlas in example clusters.

# Extended Data Figure 18

**Extended Data Fig. 18 | Analysis of the clustering result in multi-sensory presentation protocol. (a)** Spatial distribution of neurons clustered across sessions on the cortex in an example mouse. **(b)** The similarity of cluster results between different sessions. The self-similarity (diagonal elements) is dismissed and colored in white. **(c)** The similarity of sub-states, each state is divided into 2 sub-states to show that inner-state similarity is higher than inter-state similarity. **(d)** Comparison of inner-state similarity and inter-state similarity. 3 mice, each with 14 sub-states are shown. The white dot in the violin plot indicates the median of distribution. **(e)** The location of neurons from some representative clusters. **(f)** Histogram of the appearance times of each neuron in its preferred cluster. Error bars show s.e.m. n = 3 mice. The empirical result is more stable than the shuffled case.

# Extended Data Figure 19

**Extended Data Fig. 19 | Functional shift in multi-sensory presentation protocol. (a)** The representative response traces of the functional types in different sensory-modality-input trials. Functional clusters are defined within each trial type and aligned across sessions. **(b)** Example neurons that shift (bottom 2) or maintain (upper 2) their functional type. Left, The trial-averaged stimulus-triggered responses. Right, the location of the same neuron at different sessions on the UMAP visualization of FRID latent space.

# Extended Data Figure 20

**Extended Data Fig. 20 | Dynamics of functional cluster during ischemic strokes.** Two extra mice are shown. Spatial distributions of the functional atlas identified by FRID on the day before photothrombosis (baseline), 1 day and 5 days after photothrombosis. The area of infarct of the ischemic stroke is indicated by the yellow solid line. Clusters are colored based on their decoding accuracy to the visual stimuli.

# Extended Data Figure 21

**Extended Data Fig. 21 | Go/No-Go protocol. (a)** The weights of each pixel on the first 4 principal components (PCs) of the movement video. **(b)** Licking behavior of the mice in example trials. Each dot stands for a detected lick. The 4 dashed lines indicate the start and end of the cue, and the start and end of the response window. Left and right shows Go and No-go trials. Both correct and error trials are presented. **(c)** Task performance of the mice in training day 1, 3, and 6. The hit rate is the proportion of hit trials in Go trials. The correct rejection rate is the proportion of correct reject trials in all No-Go trials. **(d)** The regression coefficients of the motion components in the first 3 PCs of 15 functionomic clusters, and the explained variance of the 3 PCs of population response by the spontaneous movements. **(e)** Normalized minimal mean square error (MSE) of decoding continuous variables with functional clusters compared with randomly sampled neuron populations. Colored dots are empirical results; gray dots are randomly shuffled sampling results. **(f)** Same as **e** but for decoding accuracy of the discrete variables. **(g)** Diagram of the relationship between the motion information and the neural activity in example brain areas. Although neurons in different cortex areas are all driven by spontaneous movements, the amount of motion information they contain may differ. **(h)** The distribution of neurons in functional cluster 3 and cluster 12 on the cortex. **(i)** Quantification of the spatial separation degree (SSD) of the cue-best-coding cluster. Larger SSD indicates sparser spatial distribution. Learning increases the SSD in the task sessions. n = 3 mice, each with 20 technical repeats of FRID.

# Extended Data Figure 22

**Extended Data Fig. 22 | Spatial temporal functional map in Go/No-Go protocol. (a)** the spatial footprint of neurons in every functional cluster. **(b)** Calcium responses of neurons in each functional type. Arrows at the bottom indicate the start of each trial, the color of the arrow stands for the chord type. **(c)** Trial-averaged response of neurons in each functional cluster.

# Extended Data Figure 23

**Extended Data Fig. 23 | uPC visualization of each cluster under Go / No-go protocol.** The visualization of different clusters. In each sub panel, the left part shows the uPCs , the right part shows the corresponding trial-averaged traces, where solid lines represent correct trials and dashed lines represent incorrect trials. The two gray vertical dashed lines represent the start of the stimulus and the start of the decision window respectively. Different colors show the hit, miss, false alarm and correct rejection trials.

# Extended Data Figure 24

**Extended Data Fig. 24 | Spatial distributed cluster and its relationship to pupil size**. **a**, The spatial distribution of all 11 clusters on the CCF atlas. **b**, Left, the spatial distribution of cluster #10. Right, the normalized number of neurons in each cortical area. The normalization is done by dividing the neuron number in each cortical area by the total neuron number in that area. **c**, The variance explained by the pupil size in each functional cluster. **d**, Neural activities in cluster #10. **e**, The change of pupil size with time.

# Video S1

**Mesoscale neural recording with real-time, ultra-large-scale, high-resolution 3D mesoscope (RUSH3D).** The movie demonstrates the mesoscale neural recording technique we employed in this study. Up to tens of thousands of neurons’ activity in a 3D volume could be recorded simultaneously.
